# Differential molecular profiles and associated functionalities characterize connective tissue grafts obtained at different locations and depths in the human palate

**DOI:** 10.1038/s41368-023-00260-1

**Published:** 2023-12-11

**Authors:** Maria B. Asparuhova, Xiaoqing Song, Dominic Riedwyl, Geert van Geest, Dieter D. Bosshardt, Anton Sculean

**Affiliations:** 1 Laboratory of Oral Cell Biology, School of Dental Medicine, University of Bern, Bern, Switzerland; 2 Department of Periodontology, School of Dental Medicine, University of Bern, Bern, Switzerland; 3 Interfaculty Bioinformatics Unit, University of Bern, Bern, Switzerland; 4 Robert K. Schenk Laboratory of Oral Histology, School of Dental Medicine, University of Bern, Bern, Switzerland

**Keywords:** Growth factor signalling, Cellular signalling networks, Extracellular matrix

## Abstract

The present study aimed to assess the molecular profiles of subepithelial connective tissue grafts (CTGs) obtained at different locations and depths in the human palate. Sixty-four CTGs belonging to anterior deep (AD), anterior superficial (AS), posterior deep (PD), and posterior superficial (PS) groups were subjected to RNA-Sequencing and their transcriptomes were analyzed computationally. Functional correlations characterizing the CTG groups were validated by cell biological experiments using primary human palatal fibroblasts (HPFs) extracted from the CTGs. A clearly more pronounced location-dependent than depth-dependent difference between the grafts, with a minimal number of genes (4) showing no dependence on the location, was revealed. Epithelial, endothelial, and monocytic cell migration was strongly (*P* < 0.001) potentiated by AD- and PS-HPFs. Moreover, significantly increased expression of genes encoding C-C and C-X-C motif chemokine ligands as well as significantly (*P* < 0.01) activated p38 signaling suggested immunomodulatory phenotype for AD- and PS-HPFs. Increased growth factor gene expression and significantly activated (*P* < 0.001) Erk and Akt signaling in HPFs originating from A-CTGs implied their involvement in cell survival, proliferation, and motility. Prominent collagen-rich expression profile contributing to high mechanical stability, increased osteogenesis-related gene expression, and strongly activated (*P* < 0.001) Smad1/5/8 signaling characterized HPFs originating from P-CTGs. The present data indicate that in humans, differences between palatal CTGs harvested from different locations and depths appear to be location- rather than depth-dependent. Our findings provide the basis for future personalization of the therapeutic strategy by selecting an optimal graft type depending on the clinical indications.

## Introduction

Autologous subepithelial CTGs harvested from the patient palate are frequently used for soft tissue augmentation at natural teeth and dental implants or for pre-prosthetic reconstruction of alveolar ridge deficiencies.^1,2^ Furthermore, oral palatal full-thickness grafts are used in eyelid repair^3,4^ or eye socket reconstruction for an artificial eye.^5,6^ These applications have shown good clinical outcomes and graft survival with minor contraction and epithelial differentiation typical of the palatal donor tissue.

Histological studies have shown that healing at the gingiva-root interface following surgical soft tissue augmentation procedures often involves the formation of a long junctional epithelium with varying amounts of new connective tissue covering the root surface.^7^ This, together with changes in the width of attached keratinized mucosa, which can affect the treatment outcome,^8^ might depend on the biological properties of the autogenous CTG. It has previously been demonstrated that the specificity of the gingival connective tissue is conserved and inherited after a heterotrophic transplantation of gingiva into alveolar mucosa.^9^ Moreover, connective tissue origin was detrimental for the epithelial differentiation.^10^ Granulation tissue derived from the supra-alveolar connective tissue possessed the ability to induce the formation of keratinized epithelium whereas granulation tissue proliferating from the alveolar mucosa led to the formation of a non-keratinized epithelium.^10^ In line with these findings, a recent in vivo study has shown that following complete removal of keratinized mucosa around implants, the newly formed soft tissue barrier, similarly to alveolar mucosa, was characterized by a non-keratinized epithelium and an underlying connective tissue rich in elastic fibers.^11^ In contrast, the spontaneous regeneration at identically treated tooth sites was characterized by the formation of soft tissue resembling gingiva. These findings strongly suggest that the wound healing response and respectively, the clinical outcome depend on the biological properties of the tissues surrounding and/or underlying the defect site.

Key characteristics of experimental wounds created in the palatal keratinized masticatory mucosa are the fast healing speed and reduced scar formation as compared to cutaneous wounds.^12^ The reason for the superior healing phenotype of the palatal mucosa is not completely understood but a number of studies attribute it to a specific compilation of molecular constituents at this anatomical location.^13,14^ Furthermore, compared to skin that contains an elastic and loosely organized connective tissue, the palatal mucosa contains a denser connective tissue that directly attaches to the underlying bone, pointing to the extracellular matrix (ECM) constituents as potential mediators of the healing response.^15,16^

A number of studies focused on evaluating the wound healing response at the recipient site when utilizing subepithelial CTGs for oral soft tissue augmentation.^17–20^ It became evident that variations in the size and shape of the palatal vault affected the graft dimensions.^21,22^ However, the biological properties of the harvested CTGs have so far never been systematically evaluated. Thus, the present study aimed to assess the molecular profiles of CTGs harvested from different locations (anterior versus posterior; abbreviated A- versus P-CTGs) and depths (deep versus superficial; abbreviated D- versus S-CTGs) of the patient palate.

## Results

### RNA-Sequencing of a library of 64 CTGs reveals a significantly more pronounced location-dependent than depth-dependent difference between the grafts

**Fig. 1:**
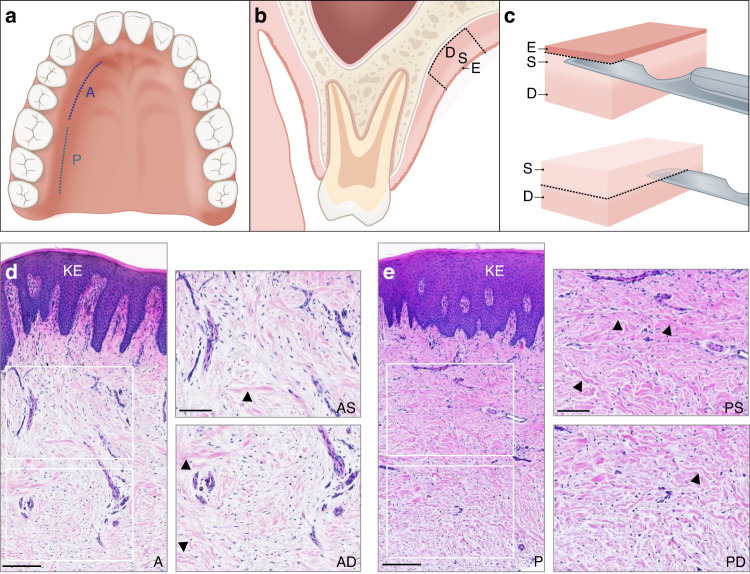
Harvesting and histological analysis of tissue samples. **a**–**c** Schematic presentation of the location (**a**) and depth (**b**) of the CTGs used in the study as well as the free gingival graft harvesting technique (**c**). A anterior, P posterior, D deep, S superficial, E epithelium. **d**, **e** Representative micrographs of A-CTG (**d**) and P-CTG (**e**). Scale bar, 200 µm. The right images in (**d**, **e**) are higher magnifications of the white rectangles and show the superficial (AS and PS) and deep (AD and PD) portions of the CTGs. Scale bar, 100 µm. Some prominent collagen fibers are designated with arrowheads. KE keratinized epithelium

Palatal CTGs from four groups (1) anterior deep (AD), (2) anterior superficial (AS), (3) posterior deep (PD), and (4) posterior superficial (PS) were obtained from 16 subjects per group as described in the Materials and Methods and depicted in Fig. 1a–c. Histological analysis of the four CTG types showed no significant morphological differences between the grafts, including no visible adipose tissue characteristics were detected (Fig. 1d, e).

**Fig. 2:**
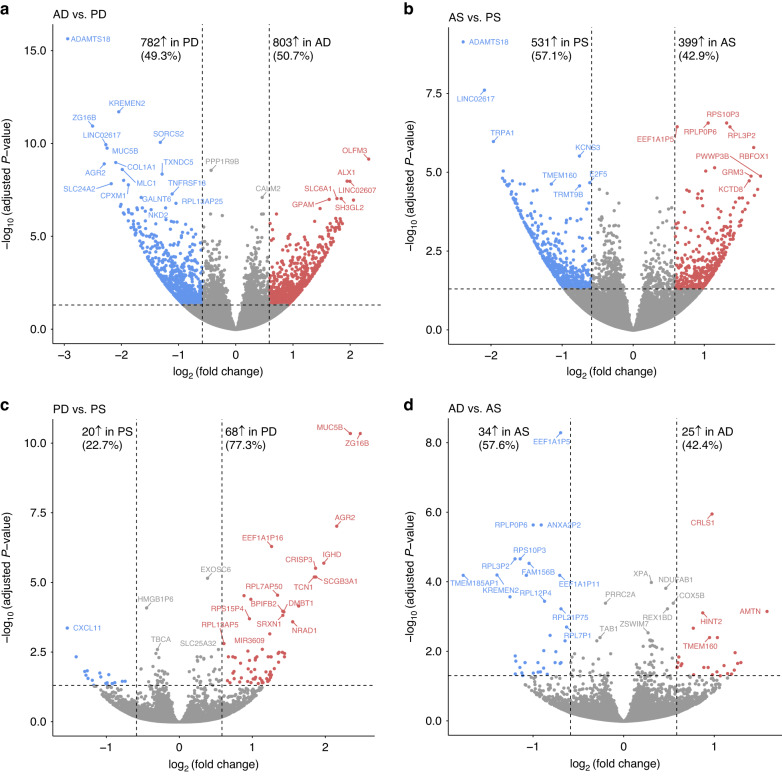
Volcano plot filtering analysis of RNA-Sequencing data. **a**–**d** Volcano plots based on the log2 fold change (x-axis) and the adjusted *p*-value in –log_10_ scale (y-axis) of all transcripts in the CTG types described in Fig. 1. Transcripts that are similarly expressed between AD and PD (**a**), AS and PS (**b**) PD and PS (**c**) and AD and AS (**d**) are shown as gray dots. The vertical lines represent 1.5 fold changes both upregulated (right side) and downregulated (left side), and the horizontal line represents an adjusted *P*-value of 0.05. All colored dots in the plots represent the differentially expressed transcripts with statistical significance in the four contrasts

By the means of Next-Generation Sequencing (NGS) technology, namely RNA-Sequencing, we obtained the transcriptomes of the collected 64 CTG samples. A volcano plot filtering analysis (Fig. 2a–d), using a minimal log2 fold change of 0.6 and a maximal *p*-value of 0.05 (presented in a –log_1__0_ scale) as the cutoff thresholds, identified a significant number of differentially expressed transcripts between the experimental groups. Complete gene lists and annotations are found in Supplementary File 1. Out of 1 585 transcripts identified as differentially expressed between A- and P-CTGs harvested in the deep region, 803 (corresponding to 50.7%) were upregulated in AD- versus 782 (corresponding to 49.3%) in PD-CTGs (Fig. 2a). Two times less differentially expressed transcripts, but similar percentage of upregulated ones, characterized the difference between A- and P-CTGs in the superficial region (Fig. 2b). Out of 930 differentially expressed transcripts, 399 (corresponding to 42.9%) were upregulated in AS- versus 531 (corresponding to 57.1%) in PS-CTGs.

The number of differentially expressed transcripts between D- and S-CTGs (Fig. 2c, d) was dramatically lower than the number of differentially expressed transcripts between A- and P-CTGs. Out of 88 differentially expressed transcripts, 68 (corresponding to 77.3%) were significantly upregulated in PD- versus 20 (corresponding to 22.7%) in PS-CTGs (Fig. 2c). Finally, out of 59 differentially expressed transcripts, 25 (corresponding to 42.4%) were upregulated in AD- versus 34 (corresponding to 57.6%) in AS-CTGs (Fig. 2d).

**Fig. 3:**
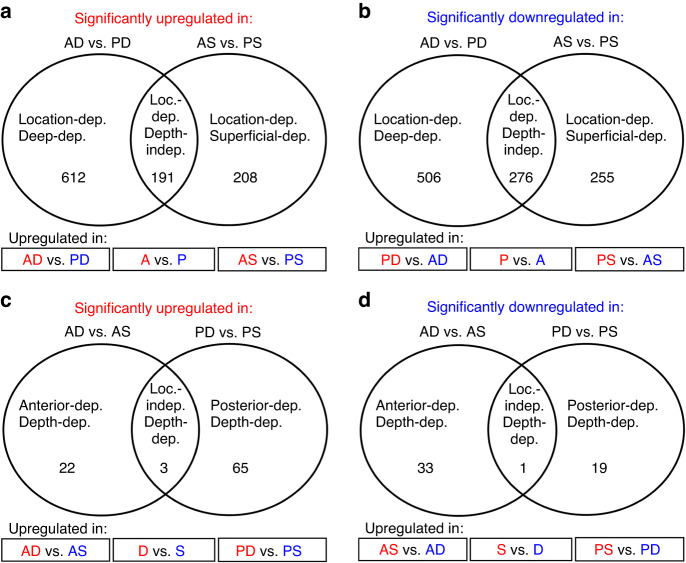
Subtractive Venn diagram analysis of RNA-Sequencing data. **a**–**d** Venn diagrams presenting classification of the genes characterizing the CTG graft types into six categories and twelve unique gene sets. The six categories are: (**a**, **b**) location-dependent/deep-dependent, location-dependent/superficial-dependent, location-dependent/depth-independent (each of the three categories has two sets for anterior- and posterior-dependent genes, shown in boxes below the Venn diagrams); (**c**, **d**) anterior-dependent/depth-dependent, posterior-dependent/depth-dependent, and location-independent/depth-dependent (each of the three latter categories has two sets for deep- and superficial-dependent genes, shown in boxes below the Venn diagrams)

The lists containing significantly up- or downregulated genes in each of the four contrasts described above were subjected to Venn diagram analysis in order to specify gene sets dependent on the location and/or depth of the graft in the human palate (Fig. 3a–d). Complete gene sets and annotations are found in Supplementary File 2. The comparison of either significantly up- or downregulated transcripts in the two contrasts AD versus PD and AS versus PS resulted in the identification of six unique gene sets. Two large sets were designated as location-dependent/deep-dependent: 1) the largest set of 612 transcripts was specifically upregulated in AD- versus PD-CTGs (Figs. 3a), and (2) the second large set of 506 transcripts was specifically upregulated in PD- versus AD-CTGs (Fig. 3b). Other two gene sets were designated as location-dependent/superficial-dependent: (1) a set of 208 transcripts that were specifically upregulated in AS- versus PS-CTGs (Figs. 3a), and (2) a set of 255 transcripts that were specifically upregulated in PS- versus AS-CTGs (Fig. 3b). Importantly, two gene sets were designated as location-dependent/depth-independent since they were found in both D- and S-CTGs (intersects; Fig. 3a, b): (1) a 191 transcript-set was specifically upregulated in A- versus P-CTGs, and (2) a larger 276 transcript-set was specifically upregulated in P- versus A-CTGs.

The comparison of either significantly up- or downregulated transcripts in the other two contrasts AD versus AS and PD versus PS resulted in the identification of another six unique gene sets. Two small sets were designated as anterior-dependent/depth-dependent: (1) a 22 transcript-set was specifically upregulated in AD- versus AS-CTGs (Figs. 3c), and (2) a slightly larger set of 33 transcripts was specifically upregulated in AS- versus AD-CTGs (Fig. 3d). Other two sets were designated as posterior-dependent/depth-dependent: (1) a set of 65 transcripts that were specifically upregulated in PD- versus PS-CTGs (Figs. 3c), and (2) a set of 19 transcripts that were specifically upregulated in PS- versus PD-CTGs (Fig. 3d). Two very small sets, represented by 3 and 1 transcripts, were designated as depth-dependent/location-independent: (1) the three transcripts were specifically upregulated in D- versus S-CTGs (Figs. 3c), and (2) a single transcript was specifically upregulated in S- versus D-CTGs (Fig. 3d).

In summary, the Venn diagram analysis allowed us to estimate precisely how many genes are dependent on each parameter, depth and location, which might influence the graft functionality. A significant number of genes (467) appeared to be independent on the depth of the graft harvest, whereas only a minimal number of genes (4) showed no dependence on the graft location.

### Differential functionalities characterize the different CTG types

**Fig. 4:**
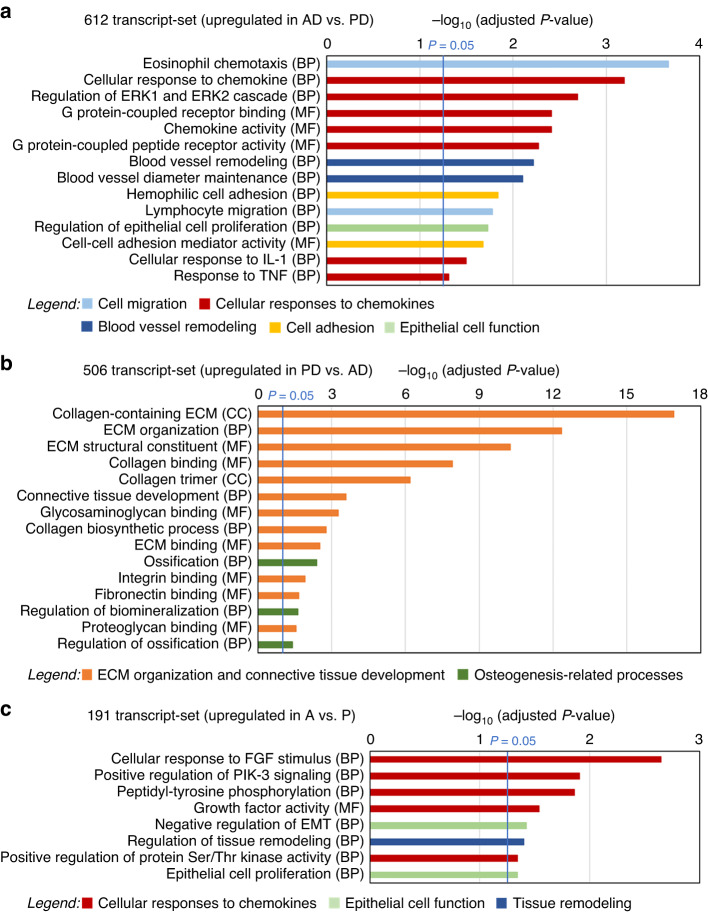
Functional correlation analysis of RNA-Sequencing data. **a**–**c** Gene ontology overrepresentation analysis of the 612 transcript-set specifically upregulated in AD- versus PD-CTGs (**a**), the 506 transcript-set specifically upregulated in PD- versus AD-CTGs (**b**) and the 191 transcript-set specifically upregulated in A- versus P-CTGs (**c**) performed with clusterProfiler. The high-level associations with biological processes (BP), molecular functions (MF), and cellular components (CC) are displayed along the x-axis of each bar chart. The y-axis displays the –log_10_ of the adjusted *P*-value. The blue vertical line denotes the cutoff for significance (*P* = 0.05). Broader functional categories combining related gene ontology terms are devised and color-coded in the legends to facilitate data interpretation

**Fig. 5:**
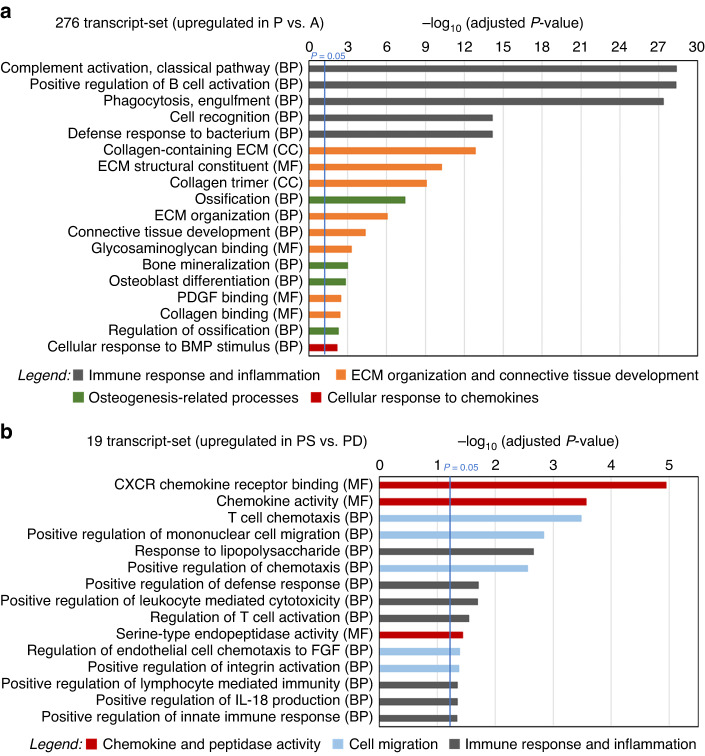
Functional correlation analysis of RNA-Sequencing data. **a**, **b** Gene ontology overrepresentation analysis of the 276 transcript-set upregulated in P- versus A-CTGs (**a**) and the 19 transcript-set upregulated in PS- versus PD-CTGs (**b**) performed with cluster Profiler. The high-level associations with biological processes (BP), molecular functions (MF), and cellular components (CC) are displayed along the *x*-axis of each bar chart. The *y*-axis displays the –log_10_ of the adjusted *P*-value. The blue vertical line denotes the cutoff for significance (*P* = 0.05). Broader functional categories combining related GO terms are devised and color-coded in the legends to facilitate data interpretation

Gene Ontology (GO) enrichment analysis of the twelve unique gene sets revealed that significantly different biological processes (BP), molecular functions (MF), and cellular components (CC) were associated with each of the sets (Figs. 4 and 5; Supplementary File 3: Fig. S1). The group of anterior-dependent/deep-dependent genes (612), upregulated in AD- versus PD-CTGs, included as major functions (1) cell migration, more specifically eosinophil and lymphocyte migration, (2) cellular responses to chemokines including regulation of extracellular signal-regulated kinases (Erk) cascades, G protein-coupled receptor binding and activity, cellular responses to interleukin-1 (IL-1) and tumor necrosis factor (TNF), (3) blood vessel remodeling, (4) cell adhesion, and (5) epithelial cell function, more specifically regulation of epithelial cell proliferation (Fig. 4a). In contrast, the group of posterior-dependent/deep-dependent genes (506), upregulated in PD- versus AD-CTGs, were associated with extremely high significance (−logp = 16.9) with collagen-containing ECM and ECM organization and connective tissue development in general (Fig. 4b). Moreover, the process of ossification was significantly (−logp ≥ 2.4) associated with this gene set.

Interestingly, some of the broader functional categories that we devised (cf. legends in Figs. 4 and 5) with the purpose to combine similar GO terms for facilitating data interpretation, were also characteristic for the two depth-independent gene sets (Figs. 4c and 5a). Thus, the group of anterior-dependent/depth-independent genes (191), upregulated in A- versus P-CTGs, included as major functions (1) cellular responses to chemokines including response to fibroblast growth factor (FGF) stimulus, positive regulation of phosphoinositide 3-kinase (PIK-3) signaling, and peptidyl-tyrosine phosphorylation, and (2) epithelial cell function such as epithelial cell proliferation and negative regulation of epithelial-to-mesenchymal transition (EMT) (Fig. 4c), suggesting a positive regulation of the opposing process of mesenchymal-to-epithelial transition (MET).^23^

The group of posterior-dependent/depth-independent genes (276), upregulated in P- versus A-CTGs, was associated with ECM organization and connective tissue development as well as osteogenesis-related processes such as osteoblast differentiation and cellular response to bone morphogenetic protein (BMP) stimulus (Fig. 5a), acting as a major regulator of the osteogenic process.^24^ Furthermore, a new functional category related to immune response and inflammation characterized this gene set with an extremely high significance (−log*P* ≥ 14.2).

Finally, the small group of posterior-dependent/superficial-dependent genes (19), upregulated in PS- versus PD-CTGs, was significantly associated with chemokine and peptidase activity, cell migration, and immune response and inflammation (Fig. 5b).

The remaining gene sets, for which the clusterProfiler analysis resulted in a significant overrepresentation of GO terms, were characterized by a single major function, e.g. transporter activity regulation was characteristic of genes upregulated in AS- versus PS-CTGs (Supplementary File 3: Fig. S1a), immune response was associated with genes upregulated in PS- versus AS-CTGs (Fig. S1b), protein folding was a major characteristic of genes upregulated in AS- versus AD-CTGs (Fig. S1c), and endocytic vesicles, as a cellular component, were mainly associated with genes upregulated in PD- versus PS-CTGs (Fig. S1d).

### Migration of epithelial, endothelial, and monocytic cells is strongly potentiated by primary HPFs with the strongest effect caused by HPFs originating from AD- and PS-CTGs

To validate the functional differences between the CTGs as suggested by the clusterProfiler analysis of the RNA-Sequencing data, we have utilized HPFs originating from the different CTG types. No significant differences between the viability and proliferative potential of HPFs obtained from AD-, AS-, PD-, and PS-CTGs were observed within the tested time periods (Supplementary File 3: Fig. S2).

**Fig. 6:**
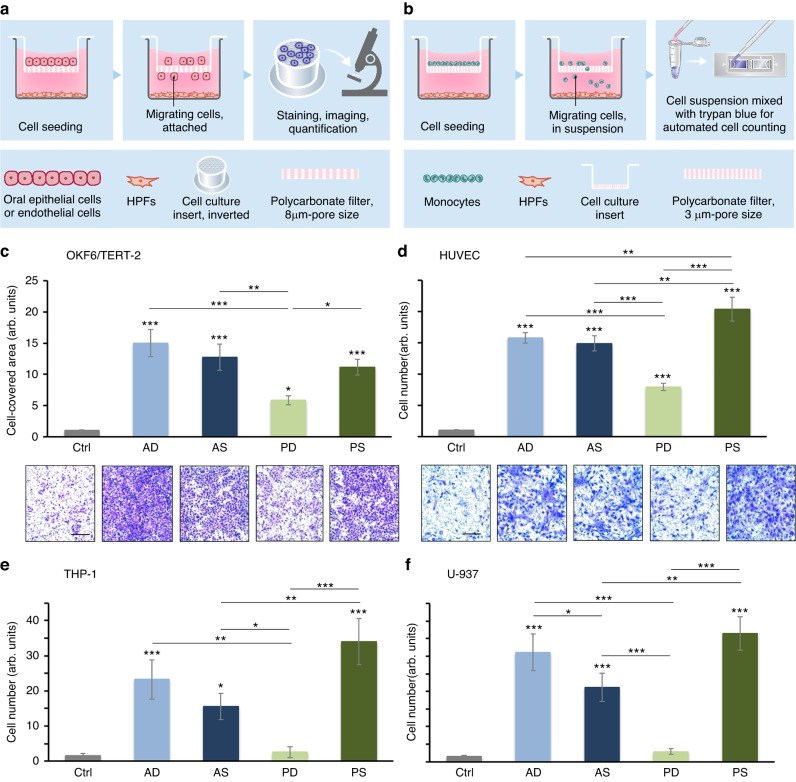
Pro-migratory effects of primary HPFs originating from AD-, AS-, PD-, and PS-CTGs. **a**, **b** Transwell migration assay setup depicting the steps of 1) cell seeding, 2) migration through polycarbonate filter insert with either 8 µm- (**a**) or 3 µm-pore size (**b**), and 3) readout for either attached oral epithelial or endothelial cells migrated on the lower side of the filter (**a**) or for migrating monocytes in suspension (**b**). For experimental details, see Materials and methods. **c**–**f** Migration of OKF6/TERT-2 (**c**), primary HUVEC (**d**), THP-1 (**e**), and U-937 (**f**) toward primary AD-, AS-, PD-, and PS-HPFs. For clarity, the abbreviation HPF is omitted and only the two letter-abbreviation (AD, AS, PD, and PS), indicating the origin of the HPF cell line from the respective CTG type is used. Bar charts present quantification of cell migration in the absence (Ctrl) or presence of HPFs by either measuring the area on the lower side of the filter covered with migrated epithelial or endothelial cells (**c**, **d**) or by automated counting of migrated monocytes in suspension (**e**, **f**). Representative images of fixed and stained cells that have migrated to the lower side of the filter in each of the experimental groups are shown (**c**, **d**). Scale bar, 500 μm. Data represent means ± SD from three independent experiments performed with three different cell donors, in duplicates. Significant differences to the control unless otherwise indicated, ***P < 0.001, **P < 0.01, *P < 0.05

The migratory capacity of two immortalized oral epithelial cell lines, hTERT TIGK and OKF6/TERT-2, primary oral human epithelial cells (HEC), primary human umbilical vein endothelial cells (HUVEC), and two monocytic cell lines THP-1 and U-937 toward each of the four HPF types was examined by using a transwell migration assay with two different readouts as depicted in Fig. 6a, b. Each of the four HPF types significantly potentiated epithelial cell migration compared to control, where the migration occurred in the absence of HPFs (Fig. 6c and Supplementary File 3: Fig. S3). The most significant stimulatory effect (*P* < 0.001) on the epithelial cell migration was exerted by AD- and AS-HPFs followed by PS-HPFs, whereas the least but still significant effect was caused by PD-HPFs. Compared to the control, the migration of HUVECs was induced by 20.4-fold by PS-HPFs, followed by AD-, AS-, and PD-HPFs (Fig. 6d). Similarly, PS-HPFs induced the strongest migratory effect on the THP-1 and U-937 monocytes, followed by AD- and AS-HPFs (Fig. 6e, f). However, PD-HPFs caused no significant migration of monocytes.

**Fig. 7:**
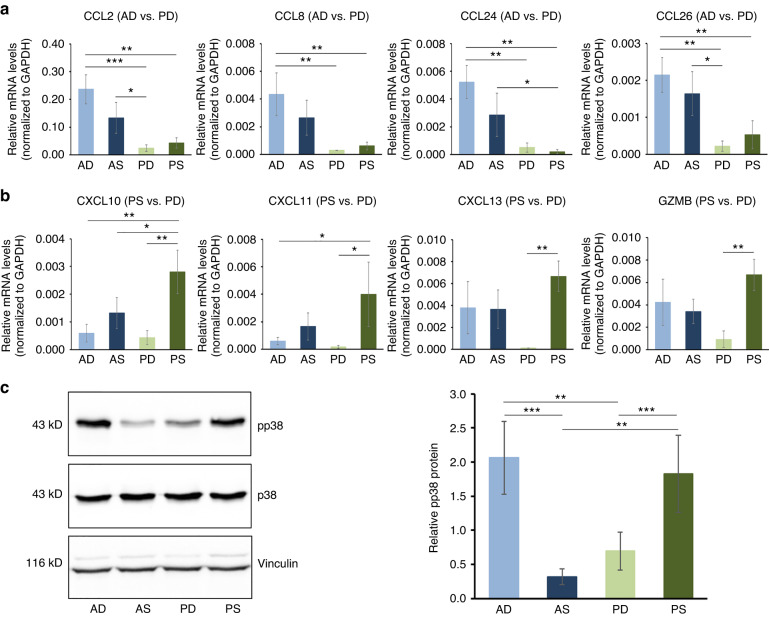
Increased expression of a number of chemokines and activated p38 kinase signaling validate the pro-migratory and immunomodulatory phenotype of AD- and PS-HPFs. **a**, **b** qRT-PCR analyses of CCL2, CCL8, CCL24, CCL26 (**a**), and CXCL10, CXCL11, CXCL13, and GZMB (**b**) transcripts normalized to GAPDH in primary AD-, AS-, PD-, and PS-HPF cells. For clarity, the abbreviation HPF is omitted and only the two letter-abbreviation (AD, AS, PD, and PS), indicating the origin of the HPF cell line from the respective CTG type is used. The affiliation of each transcript to the respective gene set is indicated in parentheses after the gene symbol. Data represent means ± SD from three independent experiments performed with three different HPF cell donors. Significant differences between experimental groups, ***P < 0.001, **P < 0.01, *P < 0.05. **c** Immunoblot analysis of phospho-p38 (pp38) protein in whole-cell extracts from primary AD-, AS-, PD-, and PS-HPFs. Blots for the total p38 protein and for the vinculin loading control are also shown. The bar chart represent densitometric quantification of the immunoblots. The pp38 levels are normalized to the total p38 protein used as an internal control. Data and statistical significance are presented as in (**a**, **b**)

The strong migration-inducing capacity of AD- and PS-HPFs was validated further by gene and protein expression analyses (Fig. 7). Four genes (CCL2, CCL8, CCL24, and CCL26) encoding C-C motif chemokine ligands showed very significant (*P* < 0.01) upregulation in AD- compared to PD- and PS-HPFs (Fig. 7a). Similarly, genes (CXCL10, CXCL11, and CXCL13) encoding C-X-C motif chemokine ligands as well as the GZMB encoding a serine protease granzyme showed significant (*P* < 0.05) upregulation in PS- compared to PD-HPFs (Fig. 7b). The gene expression analyses were further confirmed on a tissue level (Supplementary File 3: Fig. S4). Owing to strong relations of the tested genes as well as the p38 signaling pathway with cell migration, immunomodulation, and cellular responses to IL-1 and TNF,^25,26^ we investigated the activation state of the p38 kinase in HPFs. Immunoblot analysis demonstrated >5.8- and >2.6-fold increased phosphorylation levels of p38 in both AD- and PS-HPFs compared to its phosphorylation in AS- and PD-HPFs, respectively (Fig. 7c).

The increased expression of a number of chemokines and the activated p38 signaling validated the pro-migratory and immunomodulatory phenotype of AD- and PS-HPFs, which indirectly attributes the same properties to the respective CTGs.

### Increased growth factor gene expression and activated Erk and Akt signaling in HPFs originating from A-CTGs determine their role in cell survival, proliferation, and motility

**Fig. 8:**
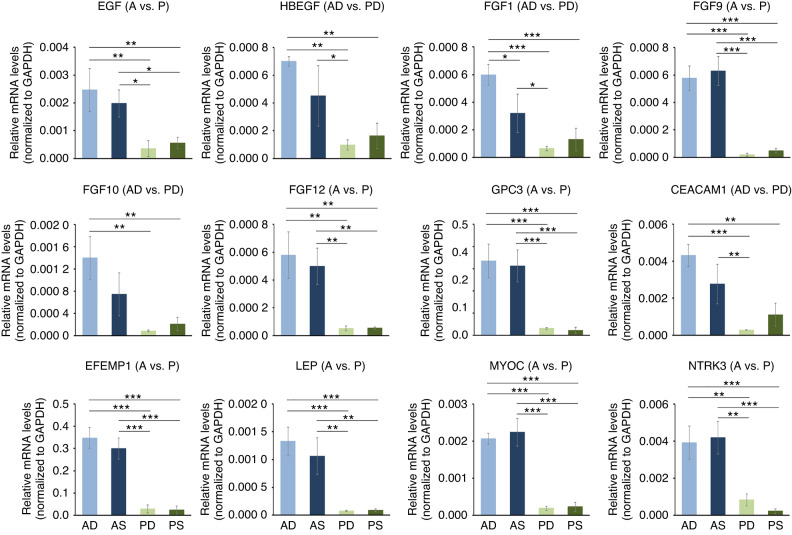
Increased growth factor gene expression in HPFs originating from A-CTGs determine their role in cell survival, proliferation, and motility. qRT-PCR analyses of EGF, HBEGF, FGF1, FGF9, FGF10, FGF12, GPC3, CEACAM1, EFEMP1, LEP, MYOC, and NTRK3 transcripts normalized to GAPDH in primary AD-, AS-, PD-, and PS-HPF cells. For clarity, the abbreviation HPF is omitted and only the two letter-abbreviation (AD, AS, PD, and PS), indicating the origin of the HPF cell line from the respective CTG type is indicated. The affiliation of each transcript to the respective gene set is indicated in parentheses after the gene symbol. Data represent means ± SD from three independent experiments performed with three different HPF cell donors. Significant differences between experimental groups, ***P < 0.001, **P < 0.01, *P < 0.05

**Fig. 9:**
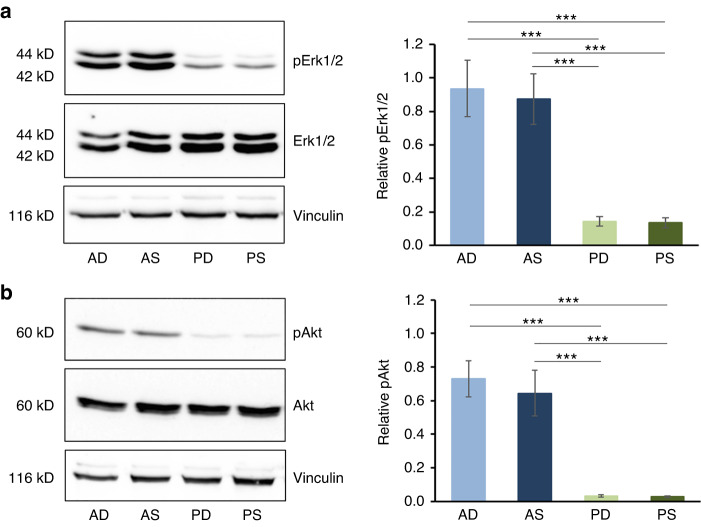
Significantly activated Erk and Akt signaling in HPFs originating from A-CTGs determine their role in cell survival, proliferation, and motility. **a**, **b** Immunoblot analyses of phospho-Erk1/2 (pErk1/2) (**a**) and phospho-Akt (pAkt) (**b**) proteins in whole-cell extracts from primary AD-, AS-, PD-, and PS-HPFs. Blots for the total Erk1/2 and Akt proteins as well as for the vinculin loading control are also shown. The bar charts represent densitometric quantification of the immunoblots. The pErk1/2 and pAkt levels are normalized to the respective total proteins used as internal controls. Data represent means ± SD from three independent experiments performed with three different HPF cell donors. Significant differences between experimental groups, ***P **<** 0.001

To validate the clusterProfiler analysis of the gene sets upregulated in A- versus P- as well as AD- versus PD-CTGs, which suggested the common association of the two sets with cellular responses to chemokines and epithelial cell function, we performed expression analysis of 12 genes (Fig. 8) and protein analysis probing two signaling pathways located downstream of the activated gene products (Fig. 9). The transcripts EGF, HBEGF, FGF1, FGF9, FGF10, and FGF12 encode growth factors, namely the epidermal growth factor (EGF), heparin binding EGF-like growth factor (HBEGF), and the fibroblast growth factors (FGF)−1, 9, 10, and 12, respectively (Fig. 8). Compared to their expression in either PD- or PS-HPFs, EGF, FGF9 and FGF12 transcripts were significantly upregulated in AD- and AS-HPFs. The HBEGF, FGF1 and FGF10 were significantly upregulated in AD- compared to PD- or PS-HPFs, whereas their expression in AS-HPFs was increased but slightly more heterogeneous. Similarly to the above listed growth factors, the remaining six transcripts, GPC3 (encoding glypican 3), CEACAM1 (encoding CEA cell adhesion molecule 1), EFEMP1 (encoding EGF-containing fibulin extracellular matrix protein 1, also known as fibulin-3), LEP (encoding leptin), MYOC (encoding myocilin), and NTRK3 (encoding neurotrophic receptor tyrosine kinase 3) were all related to regulation of cell survival, proliferation, and motility^27–32^ and were all strongly increased by several folds in AD- and AS-HPFs compared to their expression in P-HPFs (Fig. 8). The gene expression analyses were further confirmed on a tissue level (Supplementary File 3: Fig. S5).

In agreement with the gene expression data and the well-known ability of FGF and EGF growth factors to signal through the mitogen-activated protein kinase (MAPK) Erk1/2 and PI3-kinase/Akt,^33,34^ we detected increased phosphorylation of both in A-HPFs (Fig. 9a, b). In particular, the phosphorylation of the Erk1/2 in AD- and AS-HPFs was upregulated 6.0–6.9-fold compared to PD- and PS-HPFs (Fig. 9a), whereas the phosphorylation of Akt increased by 19.3–26.1-fold (Fig. 9b).

The increased expression of a number of growth factors and related molecules as well as the activated Erk and Akt signaling cascades in primary AD- and AS-HPFs indirectly validates the involvement of A-CTGs in fundamental cellular processes in response to extracellular cues, including cell survival, proliferation, migration, and differentiation.

### ECM-rich expression profile characterizes HPFs originating from P-CTGs

The GO enrichment analysis of the gene sets upregulated in P- versus A- as well as PD- versus AD-CTGs revealed a common association of the two sets with ECM organization and connective tissue development. The majority of the genes in these two sets can be clearly categorized to function in 1) building the ECM and 2) ECM remodeling and regulation (Table 1). The first category included genes encoding subunits of collagen type I, III, V, VI, X, XI, XII, XVI, XXIII, and XXVII in addition to members from the ECM structure-associated families of lecticans (ACAN, VCAN), fibrillins (FBN2), integrins (ITGA11), thrombospondins (THBS2), and tenascins (TNC). The second category included 1) genes encoding enzymes involved in the collagen biosynthetic and crosslinking processes such as lysyl oxidases (LOX and LOXL2) and prolyl hydroxylases (P3H3, P3H4), and 2) genes encoding enzymes involved in procollagen type I cleavage and matrix degradation such as the procollagen C-endopeptidase enhancer (PCOLCE), adamalysin (ADAMTS2, 3, 18) and matrix metalloproteinase (MMP2, 7, 14, 27) families.

**Table 1:** Functional categorization of genes belonging to the gene sets specifically upregulated in P- versus A- as well as in PD- versus AD-CTGs, and associated with ECM organization and connective tissue development

Gene symbol	Gene name	Gene list	Log adjusted *P*−value [PD vs. AD]	Log adjusted *P*−value [PS vs. AS]
1. Building the ECM				
**1.1 Collagens**^91,92^				
**COL1A1**	**collagen type I alpha 1 chain**	**P vs. A**	**2.1 / 1.05E-09**	**1.0 / 1.49E-02**
**COL1A2**	**collagen type I alpha 2 chain**	**P vs. A**	**1.7 / 3.57E-07**	**0.9 / 2.00E-02**
**COL3A1**	**collagen type III alpha 1 chain**	**P vs. A**	**1.4 / 8.31E-06**	**0.8 / 3.17E-02**
**COL5A1**	**collagen type V alpha 1 chain**	**PD vs. AD**	**1.4 / 2.09E-06**	
**COL5A2**	**collagen type V alpha 2 chain**	**P vs. A**	**1.1 / 1.53E-04**	**0.8 / 2.54E-02**
**COL6A1**	**collagen type VI alpha 1 chain**	**PD vs. AD**	**0.9 / 1.56E-03**	
**COL6A2**	**collagen type VI alpha 2 chain**	**PD vs. AD**	**1.0 / 1.89E-03**	
**COL10A1**	**collagen type X alpha 1 chain**	**P vs. A**	**0.9 / 4.69E-02**	**1.0 / 3.47E-02**
**COL11A1**	**collagen type XI alpha 1 chain**	**P vs. A**	**1.7 / 2.02E-06**	**1.4 / 2.84E-04**
**COL12A1**	**collagen type XII alpha 1 chain**	**PD vs. AD**	**0.8 / 2.02E-02**	
**COL16A1**	**collagen type XVI alpha 1 chain**	**PD vs. AD**	**0.9 / 9.12E-06**	
**COL23A1**	**collagen type XXIII alpha 1 chain**	**PD vs. AD**	**1.1 / 2.48E-05**	
**COL27A1**	**collagen type XXVII alpha 1 chain**	**P vs. A**	**0.7 / 1.11E-03**	**0.8 / 1.13E-03**
**1.2 Genes encoding other structural proteins**^93–97^				
**ACAN**	**aggrecan**	**PD vs. AD**	**0.9 / 9.35E-03**	
**CRISPLD2**	**cysteine rich secretory protein LCCL domain containing 2**	**PD vs. AD**	**0.9 / 6.05E-03**	
**ECM2**	**extracellular matrix protein 2**	**P vs. A**	**0.7 / 2.76E-02**	**0.7 / 3.01E-02**
**FBN2**	**fibrillin 2**	**PD vs. AD**	**1.1 / 1.98E-03**	
**ITGA11**	**integrin subunit alpha 11**	**PD vs. AD**	**1.3 / 2.48E-04**	
**MFAP2**	**microfibril associated protein 2**	**PD vs. AD**	**1.1 / 3.21E-04**	
**THBS2**	**thrombospondin 2**	**P vs. A**	**1.3 / 1.75E-04**	**1.0 / 1.12E-02**
**TNC**	**tenascin C**	**PD vs. AD**	**0.8 / 9.09E-03**	
**VCAN**	**versican**	**P vs. A**	**1.4 / 2.20E-05**	**0.9 / 1.93E-02**
2. ECM remodeling and regulation				
**2.1 Collagen biosynthetic and crosslinking processes**^98,99^				
**LOX**	**lysyl oxidase**	**P vs. A**	**0.8 / 6.89E-03**	**0.7 / 1.85E-02**
**LOXL2**	**lysyl oxidase-like 2**	**PD vs. AD**	**0.6 / 4.22E-02**	
**P3H3**	**prolyl 3-hydroxylase 3**	**PD vs. AD**	**0.6 / 1.51E-02**	
**P3H4**	**prolyl 3-hydroxylase family member 4 (inactive)**	**PD vs. AD**	**0.7 / 1.98E-04**	
**RCN3**	**reticulocalbin 3**	**PD vs. AD**	**1.1 / 2.05E-04**	
**SERPINH1**	**serpin family H member 1**	**PD vs. AD**	**0.7 / 1.15E-02**	
**2.2 Matrix degradation (including type I procollagen cleavage)**^100–102^				
**ADAMTS2**	**ADAM metallopeptidase with thrombospondin type 1 motif 2**	**PD vs. AD**	**1.2 / 5.62E-05**	
**ADAMTS3**	**ADAM metallopeptidase with thrombospondin type 1 motif 3**	**PD vs. AD**	**0.8 / 1.45E-02**	
**ADAMTS18**	**ADAM metallopeptidase with thrombospondin type 1 motif 18**	**P vs. A**	**2.9 / 2.31E-16**	**2.4 / 7.21E-10**
**PCOLCE**	**procollagen C-endopeptidase enhancer**	**P vs. A**	**1.3 / 2.13E-05**	**0.8 / 3.90E-02**
**MMP2**	**matrix metallopeptidase 2**	**PD vs. AD**	**1.1 / 9.85E-04**	
**MMP7**	**matrix metallopeptidase 7**	**PD vs. AD**	**1.1 / 8.31E-04**	
**MMP14**	**matrix metallopeptidase 14**	**PD vs. AD**	**0.7 / 1.90E-03**	
**MMP27**	**matrix metallopeptidase 27**	**PD vs. AD**	**0.7 / 3.14E-02**	

References associated with the table are found at the end of the main reference list

The table displays the fold change in logarithmic scale (logFC) and the adjusted *P*-value of the genes in each of the two contrasts, PS versus AS and/or PD versus AD

**Fig. 10:**
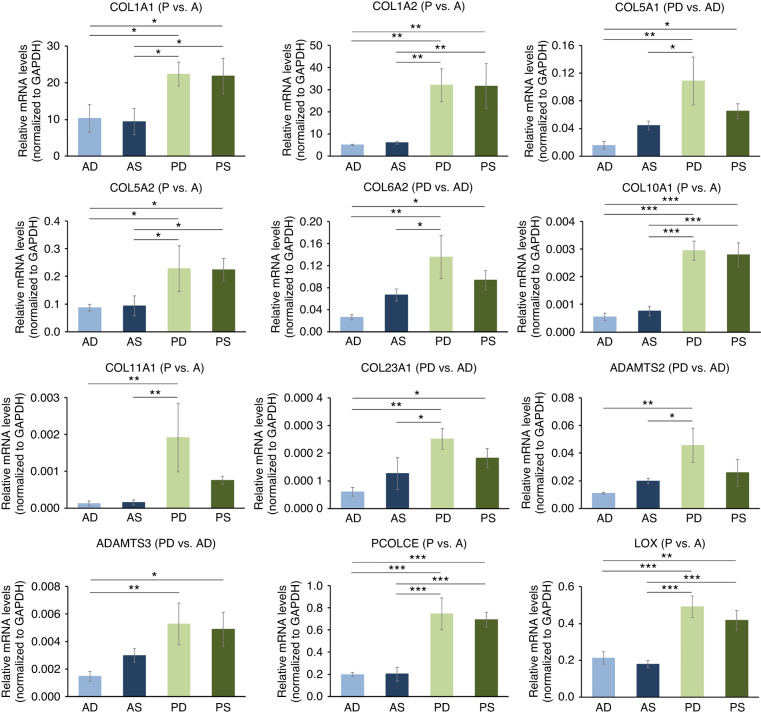
ECM-rich expression profile characterizes HPFs originating from P-CTGs. qRT-PCR analyses of COL1A1, COL1A2, COL5A1, COL5A2, COL6A2, COL10A1, COL11A1, COL23A1, ADAMTS2, ADAMTS3, PCOLCE, and LOX transcripts normalized to GAPDH in primary AD-, AS-, PD-, and PS-HPFs. For clarity, the abbreviation HPF is omitted and only the two letter-abbreviation (AD, AS, PD, and PS), indicating the origin of the HPF cell line from the respective CTG type is indicated. The affiliation of each transcript to the respective gene set is indicated in parentheses after the gene symbol. Data represent means ± SD from three independent experiments performed with three different HPF cell donors. Significant differences between experimental groups, ***P < 0.001, **P < 0.01, *P < 0.05

Many of the validated by us transcripts, namely COL1A1, COL1A2, COL5A2, COL10A1, PCOLCE, and LOX were significantly (*p* > 0.05) upregulated in the two P-HPF lines compared to their expression in A-HPFs (Fig. 10). In full agreement with the RNA-Sequencing data, COL5A1, COL6A2, COL23A1, ADAMTS2, and ADAMTS3 were significantly enriched in PD-HPFs compared to AD-HPFs. The gene expression analyses were further confirmed on a tissue level (Supplementary File 3: Fig. S6).

**Fig. 11:**
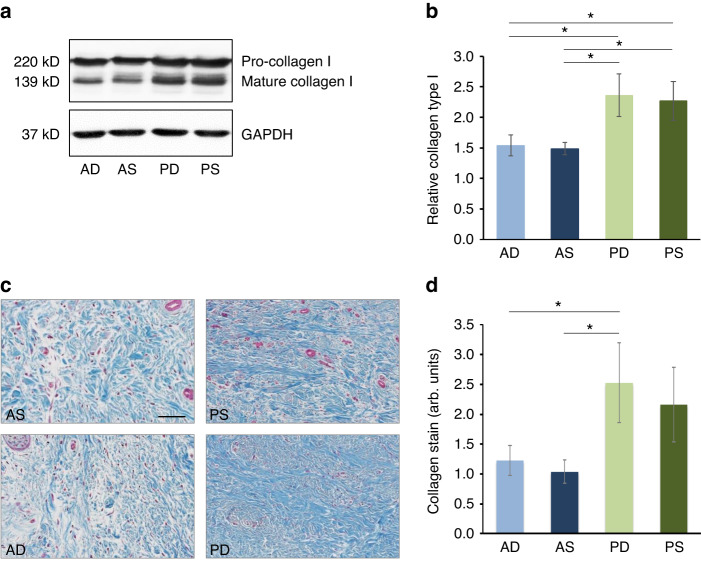
Collagen-rich expression profile characterizes P-CTGs. **a** Immunoblot analysis of pro- and mature collagen type I proteins in whole-cell extracts from primary AD-, AS-, PD-, and PS-HPFs. A blot for the GAPDH loading control is also shown. **b** Bar chart representing densitometric quantification of the immunoblots shown in (**a**). The collagen type I levels, combining both pro- and mature collagen type I, are normalized to GAPDH. Data represent means ± SD from three independent experiments performed with three different HPF cell donors. Significant differences between experimental groups, *P < 0.05. **c** Representative micrographs of AS-, AD-, PS, and PD-CTGs stained with Masson’s trichrome. Scale bar, 100 µm. **d** Bar chart representing quantification of the collagen content (blue) of the Masson’s trichrome-stained CTG samples. Data represent means ± SD from 5 samples per CTG type. Significant differences between experimental groups, *P < 0.05

Immunoblot analysis of collagen type I protein expression in HPFs revealed slight (≥1.5-fold) but significant (*p* > 0.05) enrichment of pro- and mature collagen type I in P-HPFs compared to A-HPFs (Fig. 11a, b). Furthermore, Masson’s trichrome stain of the tissue samples from each of the four CTG types confirmed the slight but significant enrichment of collagens in PD-CTGs compared to A-CTGs (Fig. 11c, d).

The collagen-rich expression profile of PD- and PS-HPFs combined with the well-known linear correlation of type I collagen deposition with mechanical stability in a range of tissues^35–37^ implies the same property to P-CTGs.

### Increased osteogenesis-related gene expression and activated Smad1/5/8 signaling in HPFs originating from P-CTGs

**Fig. 12:**
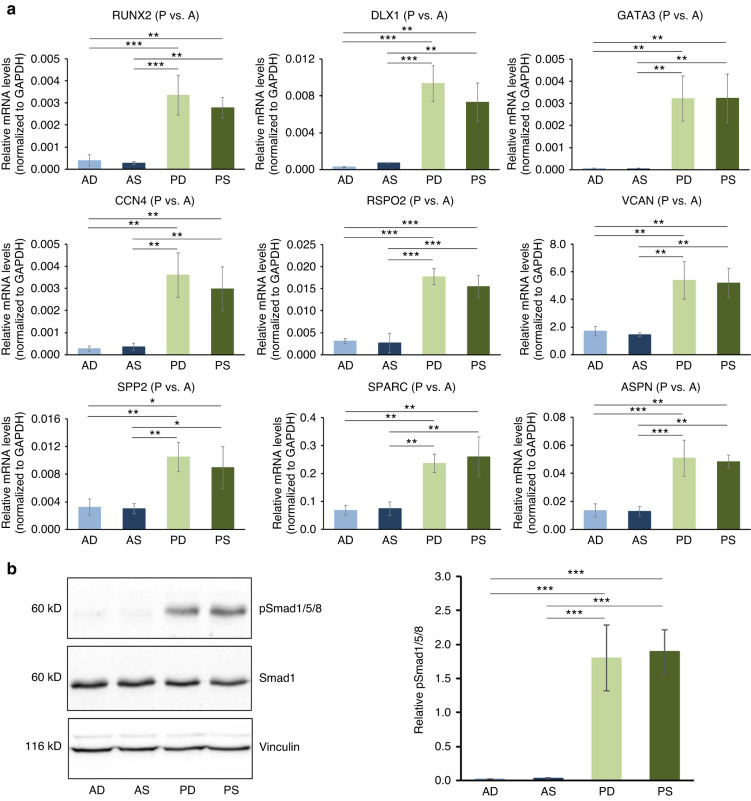
Increased osteogenesis-related gene expression and activated Smad1/5/8 signaling in HPFs originating from P-CTGs. **a** qRT-PCR analyses of RUNX2, DLX1, GATA3, CCN4, RSPO2, VCAN, SPP2, SPARC, and ASPN transcripts normalized to GAPDH in primary AD-, AS-, PD-, and PS-HPFs. For clarity, the abbreviation HPF is omitted and only the two letter-abbreviation (AD, AS, PD, and PS), indicating the origin of the HPF cell line from the respective CTG type is indicated. Data represent means ± SD from three independent experiments performed with three different HPF cell donors. Significant differences between experimental groups, ***P < 0.001, **P < 0.01, *P < 0.05. **b** Immunoblot analyses of phospho-Smad1/5/8 (pSmad1/5/8) in whole-cell extracts from primary AD-, AS-, PD-, and PS-HPFs. Blots for the total Smad1 and the vinculin loading control are also shown. The bar chart represents densitometric quantification of the immunoblots. The pSmad1/5/8 levels are normalized to the total Smad1 protein used as an internal control. Data and statistical significance are presented as in (**a**)

Finally, we validated the functional association of genes upregulated in P- versus A-CTGs with cellular responses to BMP stimulus and osteogenesis as suggested by the clusterProfiler analysis. qRT-PCR analysis of transcripts with well-known osteogenesis-related functions such as RUNX2 (encoding the runt-related transcription factor 2), DLX1 (encoding the distal-less homeobox 1), GATA3 (encoding the transcription factor GATA-binding protein 3), RSPO2 (encoding R-spondin 2), VCAN (encoding versican), SPP2 (encoding the secreted phosphoprotein 2), SPARC (encoding osteonectin), and ASPN (encoding asporin) was performed. The results showed their robust enrichment in PD- and PS-HPFs compared to the two A-HPF lines (Fig. 12a). The gene expression analyses were further confirmed on a tissue level (Supplementary File 3: Fig. S7).

Since many of the above transcripts, including RUNX2 (ref. ^38^), DLX1 (ref. ^39^), GATA3 (ref. ^40^), and CCN4 (ref. ^41^) are directly related to BMP signaling, we investigated the activation state of the transcription factors Smad1/5/8 as the pivotal intracellular effectors of BMP stimulus. Immunoblot analysis revealed >64.5-fold and >67.8-fold increased phosphorylation levels of Smad1/5/8 in PD- and PS-HPFs, respectively, compared to its phosphorylation in AD- and AS-HPFs (Fig. 12b).

## Discussion

Soft tissue augmentation in the oral cavity aims to increase tissue volume and/or width of attached keratinized mucosa in order to obtain root coverage or stable soft tissue conditions at natural teeth or dental implants.^2,42^ The present study has investigated the molecular profiles and functionalities of subepithelial CTGs harvested from different locations and depths of the human palate. Several important conclusions stemmed from our data. First, the biggest and most significant differences in the number of differentially expressed genes and associated functionalities were evident between A- and P-CTGs and less dependent on the depth of the harvest. Second, increased growth factor expression and cellular responsiveness to it via activated Erk and Akt signaling attributed involvement of A-CTGs in fundamental cellular processes such as survival, proliferation, and motility. This finding appears to suggest that the A-CTGs might be well suited for all soft tissue augmentation procedures in the oral cavity. Third, strong migration-inducing capacity of AD- and PS-HPFs for oral epithelial, endothelial, and monocytic cells, supported by increased chemokine expression and activated p38 signaling, implies pro-migratory and immunomodulatory phenotype for AD- and PS-CTGs. This finding might imply that these two graft types are especially suited for soft tissue augmentation in the esthetic zone, where fast resolution of inflammation and enhanced keratinization are of prime importance. Fourth, the prominent collagen-rich expression profile of P-CTGs linearly correlates with increased mechanical stability, which in turn suggests that these grafts are well suited for gain of tissue volume and stability around dental implants or at severe recession defects. Furthermore, osteogenesis-related profile and increased cellular responsiveness to BMP stimulus characterized the P-CTGs. This finding provides biological support for the application of P-CTGs around dental implants, where they might contribute to the establishment of both stable bone-implant and mucosa-implant interfaces.

In contrast to the clearly pronounced differences between A- and P-CTGs, our data failed to demonstrate great differences between S- and D-CTGs in terms of biological potential. Nevertheless, potential regulatory functions, such as promoter-specific protein binding and association with endocytic vesicles, described the differences between superficial and deep CTGs and deserve further investigation. In addition, the strong migration-inducing capacity of PS-CTGs compared to PD-CTGs, suggested by the clusterProfiler analysis of the RNA-Sequencing data and validated by the superior PS-HPF-induced migration of epithelial, endothelial, and monocytic cells compared to the weaker pro-migratory potential of PD-HPFs, was evident. However, our data suggested no difference in the migration-inducing potential of S- and D-CTGs harvested in the anterior palate. This is in agreement with an early histological study in humans characterizing the keratinization 3 months after transplantation of S- or D-CTGs into contralateral areas lacking keratinized mucosa.^43^ While superficial grafts displayed the histological and biochemical characteristics of keratinized mucosa (i.e. gingiva), deep grafts expressed features belonging both to keratinized and non-keratinized gingival tissues. A recent histological evaluation of keratinization 8 weeks after transplantation of S- or D-CTGs for recession coverage around teeth and implants, by the means of coronally advanced flap, in minipigs, yielded similar levels of keratinization.^44^ Notwithstanding these results, our study warrants further investigation of epithelial cell behavior under the influence of primary HPFs with A- or P-CTG origin in vitro, as well as preclinical and clinical studies investigating the speed of keratinization at defect sites healed with A- versus P-CTGs. This is conditioned not only by the clusterProfiler analysis of our RNA-Sequencing data but also by the enrichment of A-CTGs in genes with a prominent role in epithelial cell function, e.g. EGF,^45^ FGF9 (ref. ^46^), FGF10 (refs. ^47,48^), GPC3 (ref. ^49^), and EFEMP1 (ref. ^50^). Interestingly, A-CTGs were also enriched in FGFs, which play a role in cell survival, proliferation, motility, and differentiation.^51^ The impact of the A-CTGs on these processes is likely to be two-dimensional. On the one hand is the response of the cell populations, predominantly HPFs, composing the CTG itself. On the other hand, it is the response of cells composing the tissues located nearby the transplant and/or attracted to the transplant compartment that might significantly be influenced by extracellular signals released from the CTG. Signal transduction networks, such as Erk and Akt, have a common core topology irrespective of the cell type. However, the abundance of EGF and FGF receptors in the wounded tissues regenerated by an A-CTG may substantially influence the response of the healing tissue to the growth factors present in the graft. Indeed, recent data have postulated that protein abundance of Erk and Akt pathway components governs cell type-specific regulation of proliferation.^52^

Although the aim of our study, namely high-throughput molecular characterization of native CTGs, as they are harvested from the patients, was achieved in a big extent, a clear limitation is the validation of the RNA-Sequencing data in primary HPFs as the predominant cell population in the CTGs. The fact that the CTGs contain other cell populations such as adipocytes and endothelial cells, although underrepresented, should not be ignored and deserves special attention. Yet another NGS technique, namely single cell RNA-Sequencing has shown that more than one fibroblast cell population exists in the human oral mucosa.^53–55^ Using a mouse model, a recent study identified paired-related homeobox-1-positive (Prx1^+^) cells as a critical fibroblast subpopulation in anterior hard palate mucosa, which accelerated mucosal healing.^55^ Furthermore, immunofluorescent analysis of human gingival biopsies collected at various anatomical sites in the maxilla revealed that PRX1^+^ cells were present in all gingival tissues, with the highest frequency in the anterior palate rugae. A role for this fibroblast subpopulation in regulating immune response and accelerating wound healing via CCL2 among other CCL ligands was strongly suggested.^55^ This is in line with our data identifying CCL2, CCL8, CCL24, and CCL26 as being strongly enriched in HPFs originating from A- versus P-CTGs as well as with the induced monocyte migration toward A-HPFs as a source of these potent chemokines. However, our study did not identify PRX1 as differentially expressed between A- and P-CTGs most likely due to the very small size of the PRX1^+^ fibroblast subpopulation and the high number of human biopsies analyzed in our study, which reflects the human heterogeneity.

A reason for the differential molecular profile of the CTG types observed in our study may be the mixed origin (mesodermal versus neural crest) of cell populations in the hard palate. Experiments that used neural crest-specific Wnt-1 reporter animals showed that 90% of gingival connective tissue cells have a neural crest origin, whereas the remaining 10% were of mesodermal origin.^56^ Studies comparing gingival and skin fibroblasts have indeed proven that different embryonic origin may result in distinct phenotypes of the cell populations.^57,58^ Nevertheless, evidence exist that the memory of positioning and patterning, initially established during embryonic development, is modified by the local microenvironment in the tissue.^59^ Our study showed that P-CTGs are significantly enriched in collagen-containing ECM compared to A-CTGs. The specific composition of the ECM, which has the ability to bind to integrins and growth factors,^60^ will potentially influence how the cells situated in this niche will interact with it and between themselves, and what type of intracellular signaling will be activated by distinct extracellular cues. Indeed, it has been shown that a distinct set of integrin receptors are involved in the interaction of gingival cells with their specific niche.^57,61^ Whether the developmental origin of the cells composing the CTG or the local microenvironment in the CTG contributes equally to its functionality during the in vivo regeneration process remains to be investigated.

In relation to the collagen-rich expression profile of P-CTGs, it should be noted that both chains forming the major collagen type I in HPFs originating from P-CTGs were upregulated together with other collagen types such as type III,^62^ V,^63^ and XII,^64^ found to be crucial for type I collagen assembly and deposition. The linear correlation of tissue stiffness and mechanical stability with the collagen type I content^36,37^ suggests that an improved stability is to be expected with the application of P-CTGs. This, however, needs to be clinically proven. Several publications reported the appearance of bone exostosis following application of either subepithelial^65^ or free gingival^66,67^ grafts but the reason for these manifestations is not known. The current study offers a possible explanation through the prominent osteogenesis-related profile of P-CTGs. Our data suggest that for soft tissue augmentation around dental implants acquiring simultaneous bone augmentation, the combination of a P-CTG with an osteoconductive bone substitute material might be a better choice than a combination of a P-CTG with an osteoinductive bone autograft. We have previously shown that autogenous bone releases BMP-2 within relatively short periods of time.^68^ Moreover, transforming growth factor-β1 released simultaneously from the autogenous bone inhibits the BMP-2 antagonist noggin thus prolonging the BMP-activity at the augmented site.^68^ According to the present study, such prolonged BMP activity, provided by the bone autograft, might trigger prominent Smad1/5/8 signaling in the cells composing the P-CTG, thus potentially leading to the formation of bony exostosis.

To the best of our knowledge, this is the first study that identifies the transcriptomes of a high number of CTGs obtained at different locations and depths in the human palate on a genome-wide scale, and attributes differential functionality to the CTG types. In contrast, a recent study that used a targeted gene expression technique, assessing a panel of 770 genes specifically associated with fibrosis, identified no significant differences in the genetic profiles of anterior and posterior palatal samples.^69^ It should be noted, however, that the present study is not without deficiencies. A major drawback is the standardization of the graft harvesting in the superficial—deep axis. Studies have shown an extremely high inter- and intraindividual variability in terms of both palatal mucosal thickness^70–74^ and histological composition, namely fibrous connective tissue and fatty/glandular tissue proportions.^75,76^ Histological investigations of 30 human CTGs have shown that the portion of the lamina propria was in the range of 21.1–100.0 ± 25.8% (mean 65.2%) of the graft whereas the portion of the submucosa, located apical to the lamina propria and mainly composed of adipose tissue, varied between 0.0–79.0 ± 25.8% (mean 34.8%) of the graft.^75^ Despite the high interindividual variability in terms of palatal mucosal thickness and histological composition, rendering the standardization of the graft harvesting nearly impossible, we obtained high significance for the functional associations to the different CTG types. The significance was achieved both in terms of bioinformatics analysis of the RNA-Sequencing data and in validation experiments by the means of cell and molecular biology techniques. This warrants the clinical translation of our data, namely clinical trials with purposeful application of specific CTG types for specific indications with long-term clinical outcome accounting. Furthermore, analysis of the expression of the gene sets identified by us as specifically characterizing differentially localized CTG types may serve as an in vitro evaluation strategy for the suitability of novel biomaterials as soft tissue substitutes in surgical periodontal therapies.

## Materials and methods

### Tissue samples and cell culture

Palatal CTGs from the four analyzed groups AD, AS, PD, and PS were obtained from 16 systemically and periodontally healthy subjects per group. The Ethics Committee, Bern, Switzerland approved the study (ethical code ID 2018–00661 from August 13, 2018), and informed consent was obtained from all patients. The CTGs were biopsied during recession coverage surgeries performed by the same experienced periodontist (A.S.) using a standardized procedure.^77^ In agreement with a previously adopted nomenclature for palatal subepithelial CTG harvesting,^76,78^ CTGs harvested in the region of lateral incisor, canine and premolars are designated as anterior (A), whereas CTGs harvested in the molars’ area are designated as posterior (P) (Fig. 1a). At each location, the tissue sample was harvested *in toto* as a free gingival graft of ~6 mm × 4 mm (Fig. 1b, c). Any visible remnants of fat/glandular tissue were removed from the CTG. Subsequently, the epithelial layer of ~0.5 mm was removed by sharp dissection and the CTG was split into two thinner grafts—a superficial (S) located immediately subepithelially and a deep (D) located closer to the bone. Immediately after excision, the tissue sample was divided into three pieces: one was used for RNA extraction by the RNeasy Fibrous Tissue Mini Kit (Qiagen, Basel, Switzerland) as described,^79^ the second was histologically processed, and the third was used for extraction of primary HPF cells by the tissue explant technique as described.^80^ HPFs were cultured in DMEM medium (ThermoFisher Scientific, Basel Switzerland) supplemented with 10% fetal calf serum (FCS; ThermoFisher Scientific) and 1% antimycotics/antibiotics (AA; ThermoFisher Scientific). Primary HECs were extracted from epithelial samples by the tissue explant technique and propagated in complete Keratinocyte serum-free medium (KSFM; ThermoFisher Scientific) supplemented with 0.2 ng·mL^−1^ human recombinant EGF (Peprotech, London, UK), 25 μg·mL^−^^1^ bovine pituitary extract (ThermoFisher Scientific), and 1% AA. The immortalized oral epithelial cell lines hTERT TIGK (ATCC, Manassas, VA, USA) and OKF6/TERT-2 (ref. ^81^) were cultured in complete KSFM. Primary HUVECs (ATCC) were propagated in complete M199 medium as described.^82–86^ The human monocytic THP-1 and U-937 cell lines (both from ATCC) were propagated in RPMI-1640 medium supplemented with 10% FCS and 1% AA. All cell types (passages 1–5 for the primary cells) were starved in 0.3% FCS/DMEM before culturing under experimental conditions.

### Histological processing

Tissue samples were fixed in 4% buffered formaldehyde (Sigma, Basel, Switzerland) for 4 days and decalcified in 4.13% ethylenediaminetetraacetic acid (Sigma) for 7 days before paraffin-embedding. Subsequently, 5 µm-tick tissue sections were stained with hematoxylin/eosin (Sigma) and Masson’s trichrome (Sigma), and imaged on an Axio Imager M2 microscope (Carl Zeiss, Oberkochen, Germany) equipped with an AxioCam MRc camera (Carl Zeiss). Collagen content was quantified by using the Fiji distribution of ImageJ.

### RNA-Sequencing and bioinformatics analysis

Quantity and quality of RNA extracted from each of the 64 CTGs were assessed using the Qubit RNA BR assay on a Qubit 4.0 fluorometer (ThermoFisher Scientific) as well as the Fragment Analyzer RNA kit on an Advanced Analytical Fragment Analyzer (Agilent Technologies, Basel, Switzerland). Sequencing libraries were produced using the TruSeq Stranded mRNA Library Prep kit in combination with TruSeq RNA UD Indexes according to Illumina’s guidelines (Illumina, San Diego, CA, USA). Pooled cDNA libraries were sequenced paired-end using the NovaSeq 6000 S2 Reagent Kit v1.5 (200 cycles) on a NovaSeq 6000 instrument (Illumina), generating an average of 37 million reads/sample. The quality of the sequencing run was assessed using Sequencing Analysis Viewer v2.4.7 software and all base call files were demultiplexed and converted into FASTQ files using bcl2fastq Conversion Software v2.20 (Illumina). RNA-Sequencing was performed at the Next-Generation Sequencing Platform, University of Bern, Switzerland.

The quality of the RNA-Sequencing data was further assessed using FastQC v0.11.9 and RSeQC v4.0.0 (ref. ^83^). The reads were mapped to the reference genome (GRCh38) using HISAT2 v2.2.1 (ref. ^84^). FeatureCounts v2.0.1 (ref. ^85^) was used to count the number of reads overlapping with each gene as specified in the genome annotation (Ensembl version 104). The Bioconductor package DESeq2 (ref. ^86^) was used to test for differential gene expression between the experimental groups. To be considered as differentially expressed between AD and PD, AS and PS, PD and PS, and AD and AS, genes had to pass the filters: adjusted *P*-value ≤ 0.05 (with Benjamin-Hochberg false discovery correction) and a minimal log2 fold change of 0.6. Using the above parameters, lists of up- and downregulated genes of the contrasts 1) AD versus PD and AS versus PS, and 2) AD versus AS and PD versus PS were compared resulting in the formation of twelve gene sets (Fig. 3a–d). Gene ontology enrichment (overrepresentation) analysis was performed using the clusterProfiler.^87^ All analyses were run in R version 4.1.0. RNA-Sequencing data are available from ArrayExpress, accession number E-MTAB-13141.

### Cell viability and proliferation assays

Viability of primary HPFs was assessed by the CellTiter-Blue viability assay (Promega, Madison, WI, USA) following the manufacturer’s instructions. Cellular proliferation rates were determined using BrdU ELISA (Roche, Basel, Switzerland) as described.^88^ Experimental values were normalized to the lowest value measured at the time point 0 for each of the HPF cell types. For both assays, data represent means ± SD from three independent experiments performed with three different HPF cell donors.

### Cell migration assay

Cell migration was assayed using transwell polycarbonate membrane inserts (Corning, Amsterdam, The Netherlands) with 8 µm-pore size for all epithelial and endothelial cell lines, and 3 µm-pore size for the monocytic lines (Fig. 6a, b). After 24 h of starvation, 6 × 10^4^ epithelial or endothelial cells or 1 ×10^6^ monocytes were plated in the top insert chamber in the respective serum-free medium. 1.2 × 10^4^ HPFs of each type were seeded in the lower chamber in 10% FCS/DMEM. Epithelial and endothelial cells were allowed to migrate across the filter for 18 h before fixation and crystal violet-staining. Images were acquired on an Olympus BX-51 microscope. Migration was quantified by using the Fiji distribution of ImageJ. Monocytes were allowed to migrate across the filter for 3 h, followed by collection of the migrated suspension cells and trypan blue dye-exclusion cell counting performed in a Countess™ II instrument (Invitrogen). Data represent means ± SD from three independent experiments, each performed in triplicates.

### qRT-PCR

Total RNA from HPFs was isolated using the RNeasy Mini Kit (Qiagen) and reverse transcribed using the High-Capacity cDNA RT Kit (Applied Biosystems, Rotkreuz, Switzerland) as described.^89^ qPCR for 41 different genes was carried out using the FastStart Universal SYBR Green Master ROX (Roche) on a QuantStudio 3 instrument (Applied Biosystems). Data, normalized to GAPDH, were analyzed by the ∆Ct method. Data represent means ± SD from three independent experiments performed with three different HPF cell donors. Lists with gene symbols, gene names, accession numbers, and primer sequences are found in Supplementary File 3, Tables S1–S4.

### Immunoblotting

Whole-cell extracts from HPFs were prepared by lysis in RIPA buffer as described.^90^ Lysates were run on either 7.5% or 10% SDS–PAGE, according to the size of the tested proteins, and transferred to Amersham™ Protran® membrane (Sigma). Proteins of interest were visualized using anti-phospho-p38 and anti-p38 (Cell Signaling Technology, Danvers, MA, USA), anti-phospho-Erk1/2 and anti-Erk1/2 (Cell Signaling Technology), anti-phospho-Akt and anti-Akt (Cell Signaling Technology), anti-collagen I (abcam, Cambridge, UK), anti-phospho-Smad1/5/8 and anti-Smad1 (Cell Signaling Technologies), anti-vinculin (Sigma), and anti-GAPDH (abcam) antibodies followed by horseradish peroxidase-conjugated secondary antibodies (MP Biomedicals, Santa Ana, CA, USA) for detection with the SuperSignal™ West Dura (ThermoFisher Scientific). The expression of the collagen type I or the phospho-proteins was quantified relative to the expression of the loading control or respective total proteins by densitometry using ImageJ. Data represent means ± SD from three independent experiments with three different HPF cell donors.

### Statistical analysis

The statistical analyses were carried out using GraphPad InStat Software v3.05. Differences between groups were assessed by one-way analysis of variance (ANOVA) with Tukey’s post hoc test. Values of *P* < 0.05 were considered statistically significant.

### Supplementary information

Supplementary File 1

Supplementary File 2

Supplementary File 3

## Data Availability

All data generated and analyzed during this study are included in this article. NGS data have been deposited in the ArrayExpress repository (https://www.ebi.ac.uk/biostudies/arrayexpress) under the accession number E-MTAB-13141.
