# Supplementary material for: Differential molecular profiles and associated functionalities characterize connective tissue grafts obtained at different locations and depths in the human palate
Source: Int J Oral Sci. 2023 Dec 11;15:57. doi: 10.1038/s41368-023-00260-1 (PMC10711016; doi:10.1038/s41368-023-00260-1)
Supplement: Supplementary file 3 [file 41368_2023_260_MOESM3_ESM.pdf]

Supplementary File 3 for:

Differential molecular profiles and associated functionalities characterize connective tissue grafts obtained at different locations and depths in the human palate

Maria B. Asparuhova[1,2*] | Xiaoqing Song[1,2] | Dominic Riedwyl[1,2] | Geert van Geest[3] | Dieter D. Bosshardt[4,2] | Anton Sculean[2]

[1]Laboratory of Oral Cell Biology, School of Dental Medicine, University of Bern, Bern, Switzerland

[2]Department of Periodontology, School of Dental Medicine, University of Bern, Bern, Switzerland

[3]Interfaculty Bioinformatics Unit, University of Bern, Bern, Switzerland

[4]Robert K. Schenk Laboratory of Oral Histology, School of Dental Medicine, University of Bern, Bern, Switzerland

***Corresponding author:** Maria B. Asparuhova
Laboratory of Oral Cell Biology
School of Dental Medicine, University of Bern
Freiburgstrasse 3, CH-3010 Bern, Switzerland
Tel: +41 31 684 05 97
Email: mariya.asparuhova@unibe.ch

Contents

Figure S1: Gene ontology overrepresentation analysis of the 208 transcript-set upregulated in AS- versus PS-CTGs (**a**), the 255 transcript-set upregulated in PS- versus AS-CTGs (**b**), the 33 transcript-set upregulated in AS- versus AD-CTGs (**c**), and the 65 transcript-set upregulated in PD- versus PS-CTGs (**d**) using the clusterProfiler.

Figure S2: Cell viability (**a**) and proliferation (**b**) of primary AD-, AS-, PD-, and PS-HPF cells originating from different CTG types.

Figure S3: Pro-migratory effects of primary HPFs originating from AD-, AS-, PD-, and PS-CTGs.

Figure S4: Increased expression of a number of chemokines validate the pro-migratory and immunomodulatory phenotype of AD- and PS-CTGs.

Figure S5: Increased growth factor gene expression in A-CTGs determine their role in cell survival, proliferation, and motility.

Figure S6: ECM-rich expression profile characterizes P-CTGs.

Figure S7: Increased osteogenesis-related gene expression in P-CTGs.

Figure S8 and S9: Uncropped immunoblots for various proteins investigated in the study.

Table S1: Primer sequences for genes belonging to the gene sets specifically upregulated in AD- versus PD- as well as in PS- versus PD-CTGs, and related to cell migration and immunomodulation.

Table S2: Primer sequences for genes belonging to the gene sets specifically upregulated in A- versus P- as well as in AD- versus PD-CTGs, and related to Erk and Akt signaling.

Table S3: Primer sequences for genes belonging to the gene sets specifically upregulated in P- versus A- as well as in PD- versus AD-CTGs, and related to ECM organization and connective tissue development.

Table S4: Primer sequences for genes belonging to the gene set specifically upregulated in P- versus A-CTGs and related to osteogenesis.

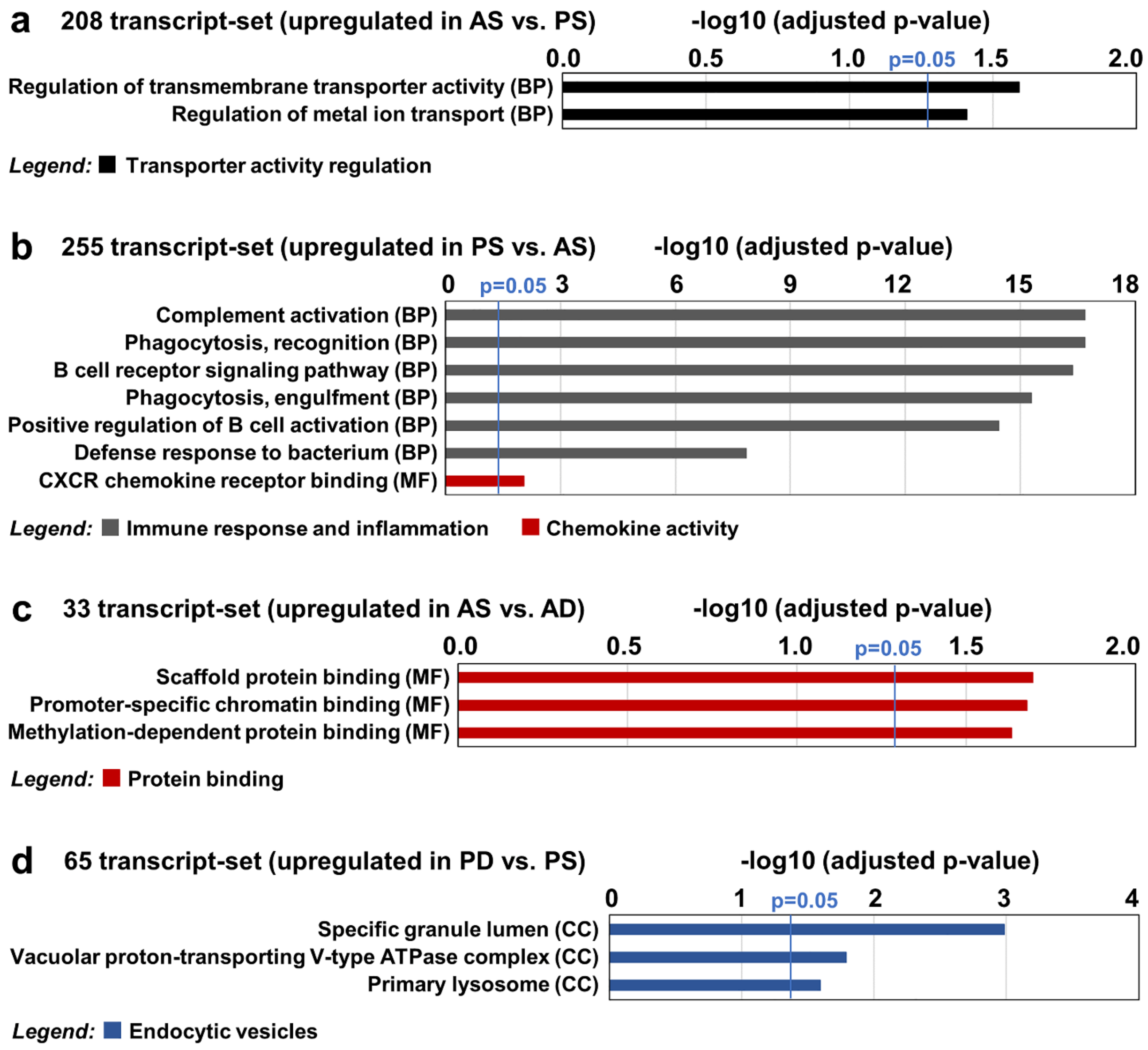

Figure S1. Gene ontology overrepresentation analysis of the 208 transcript-set upregulated in AS- versus PS-CTGs (**a**), the 255 transcript-set upregulated in PS- versus AS-CTGs (**b**), the 33 transcript-set upregulated in AS- versus AD-CTGs (**c**), and the 65 transcript-set upregulated in PD- versus PS-CTGs (**d**) using the clusterProfiler. The high-level associations with biological processes (BP), molecular functions (MF), and cellular components (CC) are displayed along the x-axis of each bar chart. The y-axis displays the –log10 of the adjusted p-value. The blue vertical line denotes the cutoff for significance (p = 0.05). Broader functional categories combining related gene ontology terms are devised and color-coded in the legends to facilitate data interpretation.

a

b

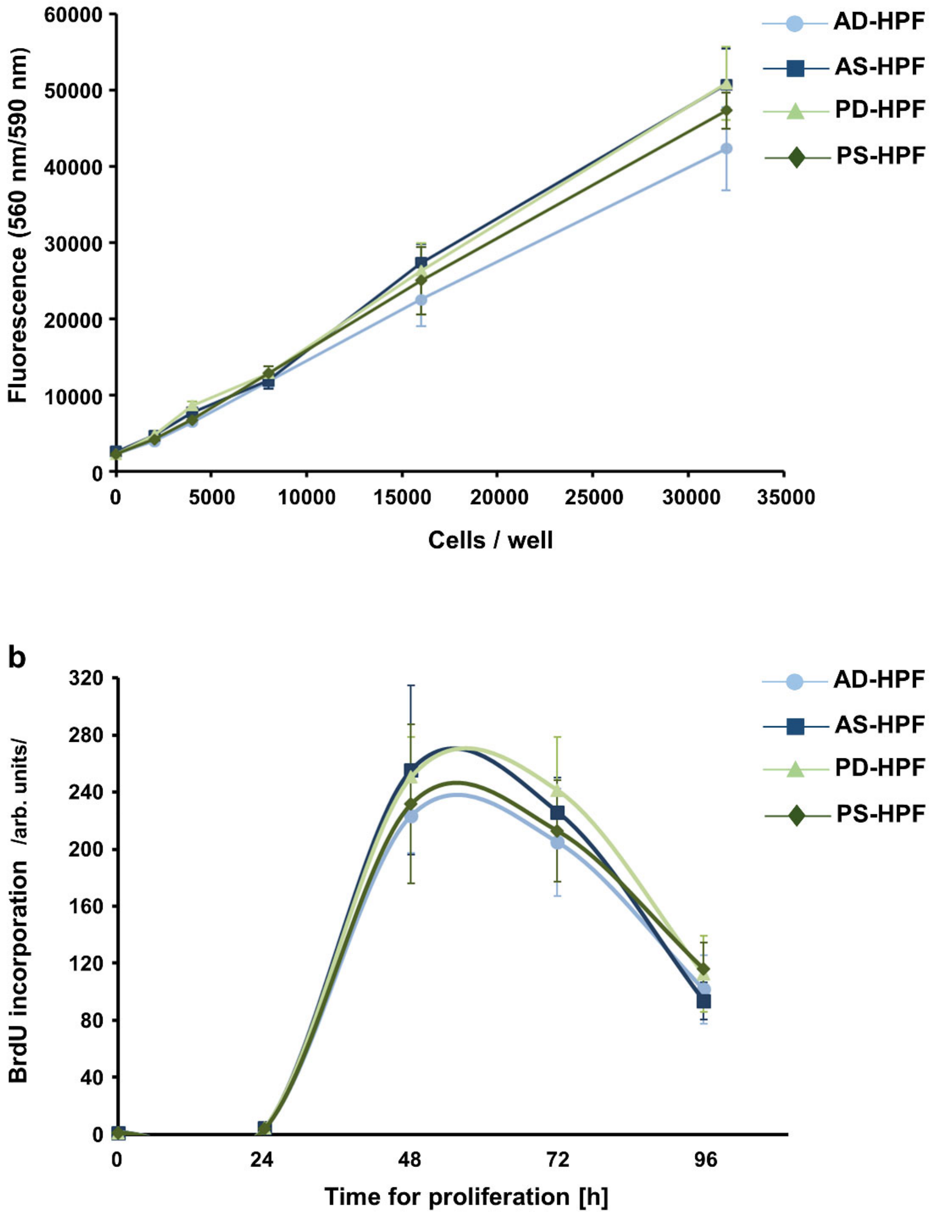

Figure S2. No significant differences in cell viability (**a**) and proliferation (**b**) of primary AD-, AS-, PD-, and PS-HPF cells originating from different CTG types. Primary HPFs of each type were subjected to the CellTiter-Blue cell viability assay (**a**) and the BrdU Cell Proliferation ELISA (**b**) according to the manufacturers' protocols and as described in the Materials and Methods section in the main text. Data represent means ± SD from three independent experiments performed with three different HPF cell donors.

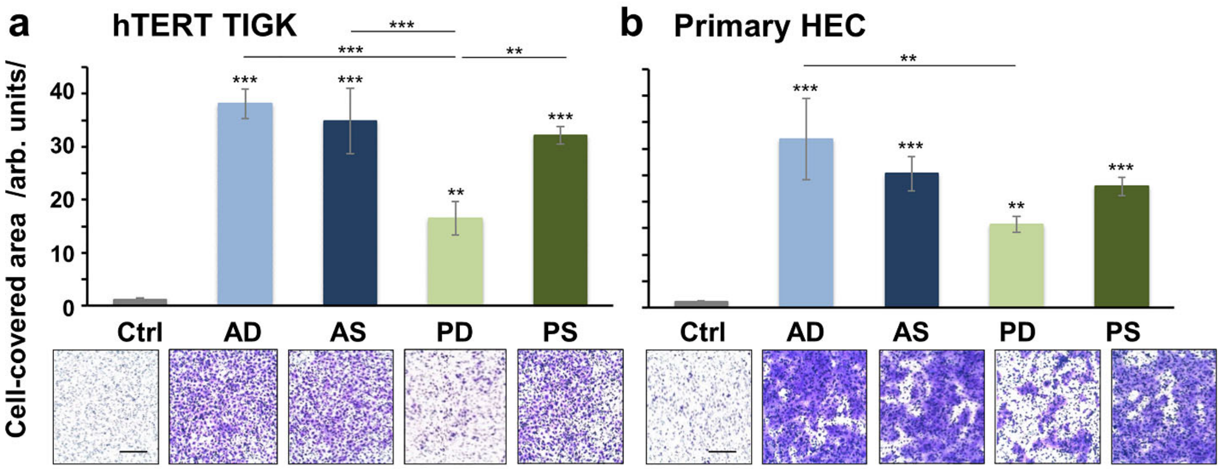

Figure S3. Pro-migratory effects of primary HPFs originating from AD-, AS-, PD-, and PS-CTGs. Migration of hTERT TIGK (**a**) and primary HEC (**b**) toward primary AD-, AS-, PD-, and PS-HPFs. For clarity, the abbreviation HPF is omitted and only the two letter-abbreviation (AD, AS, PD, and PS), indicating the origin of the HPF cell line from the respective CTG type is used. Bar charts present quantification of cell migration in the absence (Ctrl) or presence of HPFs by measuring the area on the lower side of the filter covered with migrated epithelial cells. Representative images of fixed and stained cells that have migrated to the lower side of the filter in each of the experimental groups are shown (**a, b**). Scale bar, 500 µm. Data represent means ± SD from three independent experiments performed with three different cell donors, in duplicates. Significant differences to the control unless otherwise indicated, ***$p < 0.001$, **$p < 0.01$.

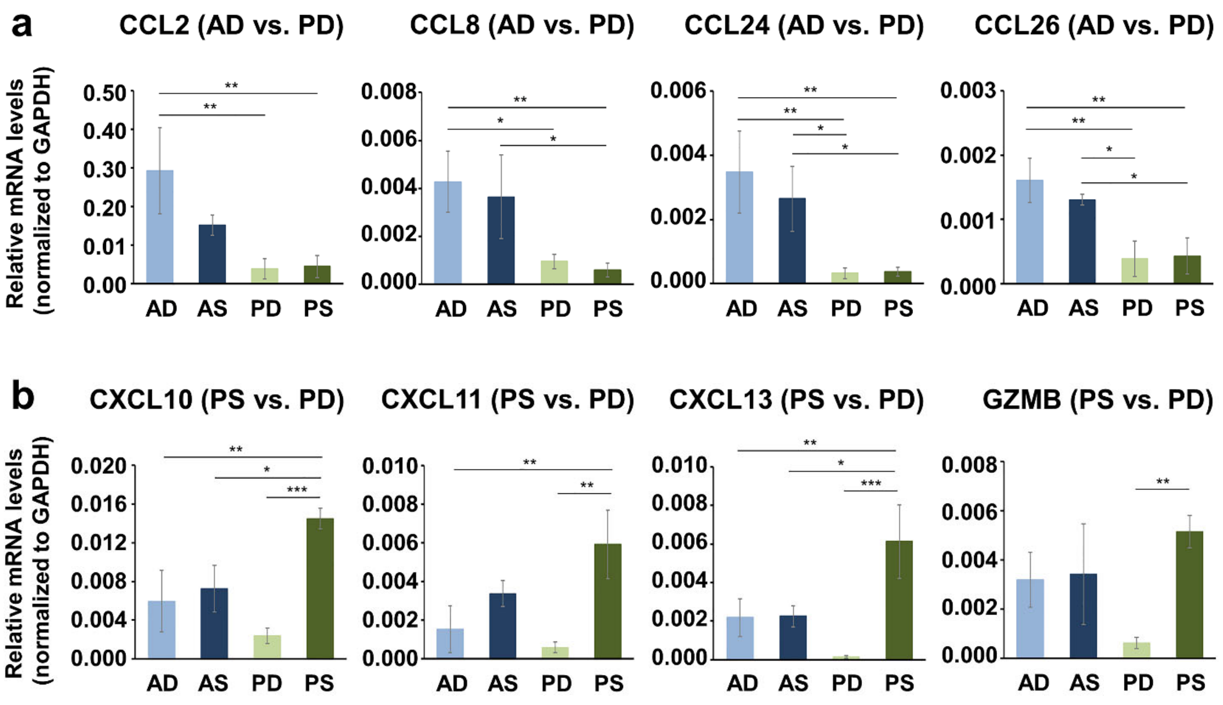

Figure S4. Increased expression of a number of chemokines validate the pro-migratory and immunomodulatory phenotype of AD- and PS-CTGs. qRT-PCR analyses of CCL2, CCL8, CCL24, CCL26 (**a**), and CXCL10, CXCL11, CXCL13, and GZMB (**b**) transcripts normalized to GAPDH in AD-, AS-, PD-, and PS-CTG tissue samples. For clarity, the abbreviation CTG is omitted and only the two letter-abbreviation (AD, AS, PD, and PS), indicating the type of the CTG, is used. The affiliation of each transcript to the respective gene set is indicated in parentheses after the gene symbol. Data represent means ± SD from 16 samples per CTG type. Significant differences between experimental groups, ***$p < 0.001$, **$p < 0.01$, *$p < 0.05$.

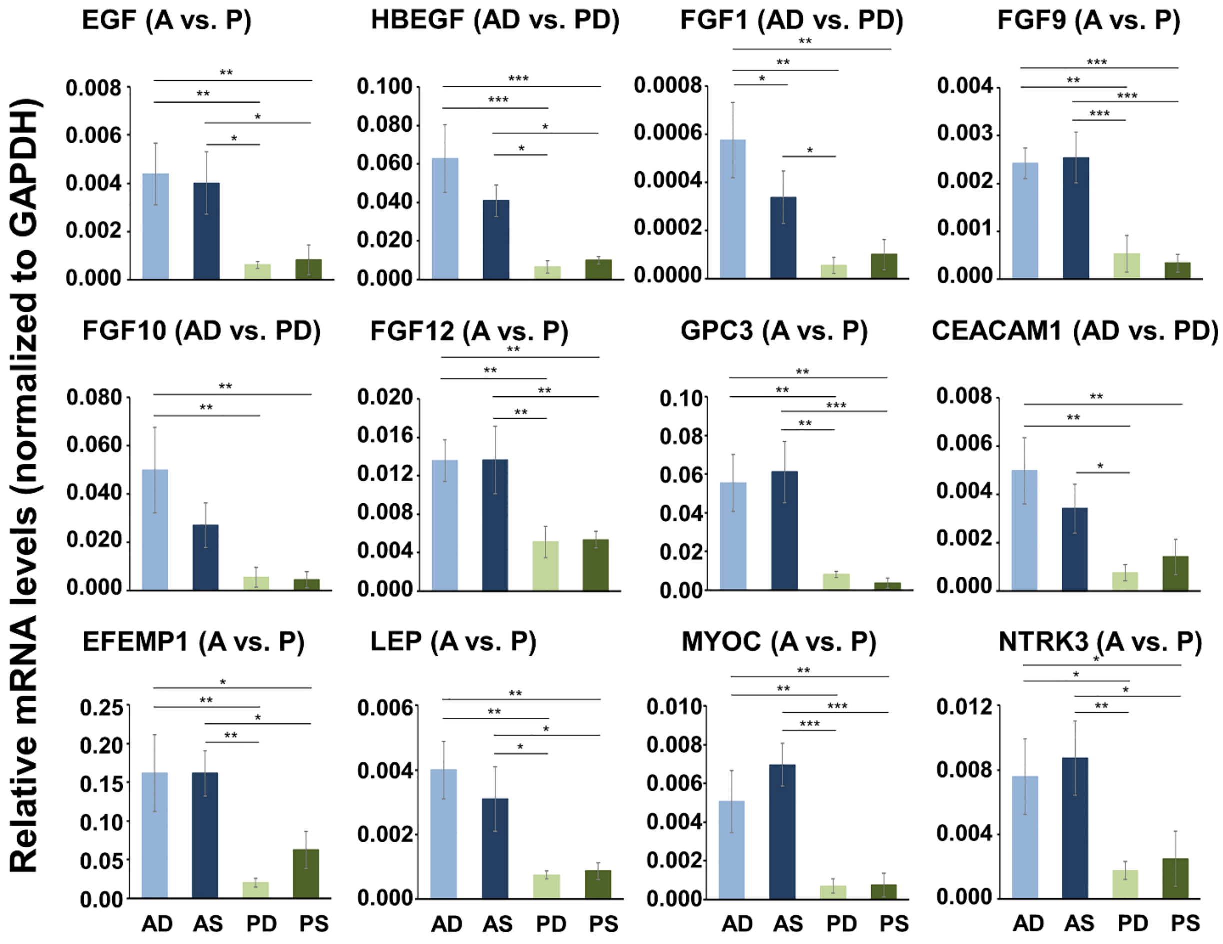

Figure S5. Increased growth factor gene expression in A-CTGs determine their role in cell survival, proliferation, and motility. qRT-PCR analyses of EGF, HBEGF, FGF1, FGF9, FGF10, FGF12, GPC3, CEACAM1, EFEMP1, LEP, MYOC, and NTRK3 transcripts normalized to GAPDH in AD-, AS-, PD-, and PS-CTG tissue samples. For clarity, the abbreviation CTG is omitted and only the two letter-abbreviation (AD, AS, PD, and PS), indicating the type of the CTG, is used. The affiliation of each transcript to the respective gene set is indicated in parentheses after the gene symbol. Data represent means ± SD from 16 samples per CTG type. Significant differences between experimental groups, ***$p < 0.001$, **$p < 0.01$, *$p < 0.05$.

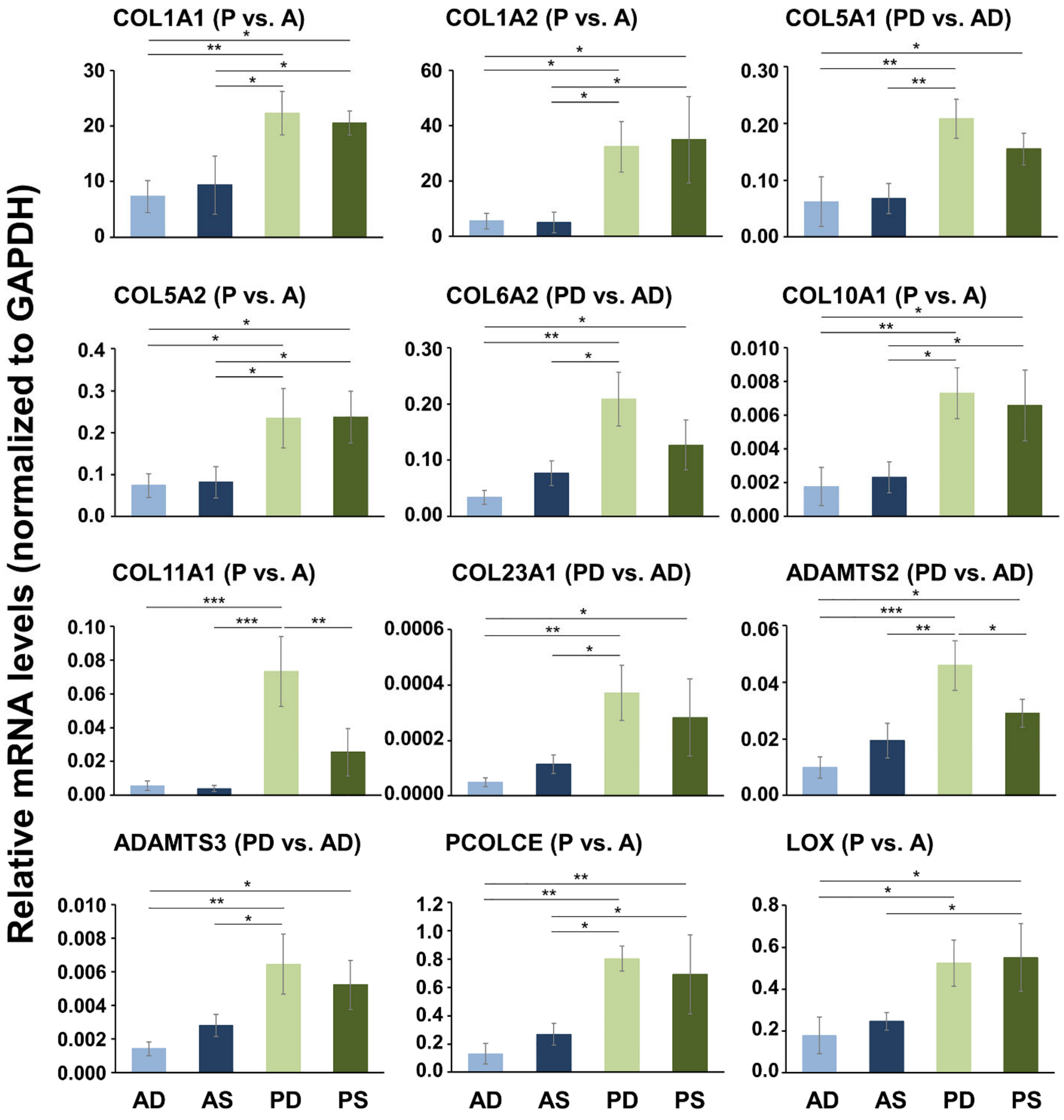

Figure S6. ECM-rich expression profile characterizes P-CTGs. qRT-PCR analyses of COL1A1, COL1A2, COL5A1, COL5A2, COL6A2, COL10A1, COL11A1, COL23A1, ADAMTS2, ADAMTS3, PCOLCE, and LOX transcripts normalized to GAPDH in AD-, AS-, PD-, and PS-CTG tissue samples. For clarity, the abbreviation CTG is omitted and only the two letter-abbreviation (AD, AS, PD, and PS), indicating the type of the CTG, is used. The affiliation of each transcript to the respective gene set is indicated in parentheses after the gene symbol. Data represent means ± SD from 16 samples per CTG type. Significant differences between experimental groups, ***p < 0.001, **p < 0.01, *p < 0.05.

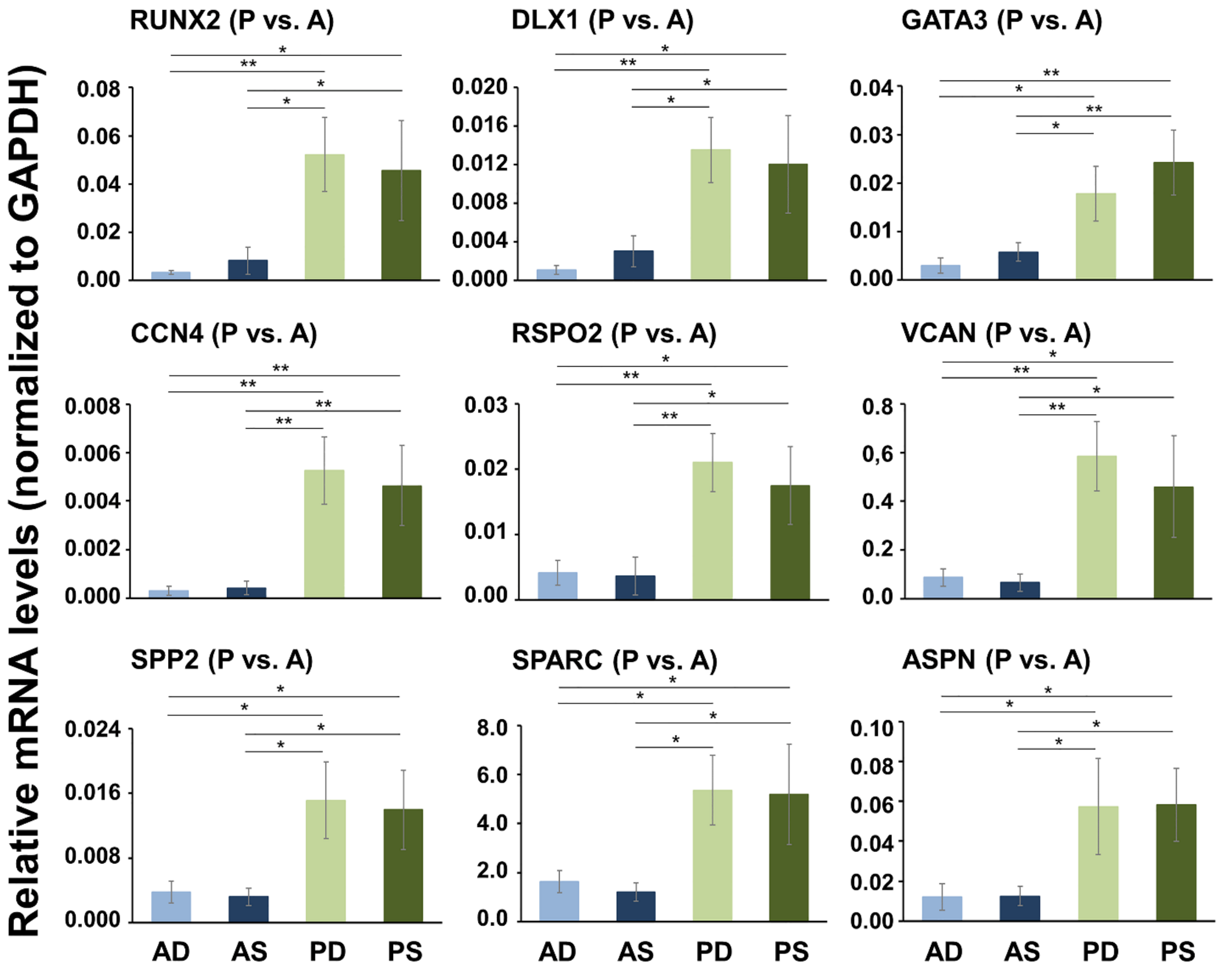

Figure S7. Increased osteogenesis-related gene expression in P-CTGs. qRT-PCR analyses of RUNX2, DLX1, GATA3, CCN4, RSPO2, VCAN, SPP2, SPARC, and ASPN transcripts normalized to GAPDH in AD-, AS-, PD-, and PS-CTG tissue samples. For clarity, the abbreviation CTG is omitted and only the two letter-abbreviation (AD, AS, PD, and PS), indicating the type of the CTG, is used. The affiliation of each transcript to the respective gene set is indicated in parentheses after the gene symbol. Data represent means ± SD from 16 samples per CTG type. Significant differences between experimental groups, **p < 0.01, *p < 0.05.

a **Uncropped blot for Fig. 5c**

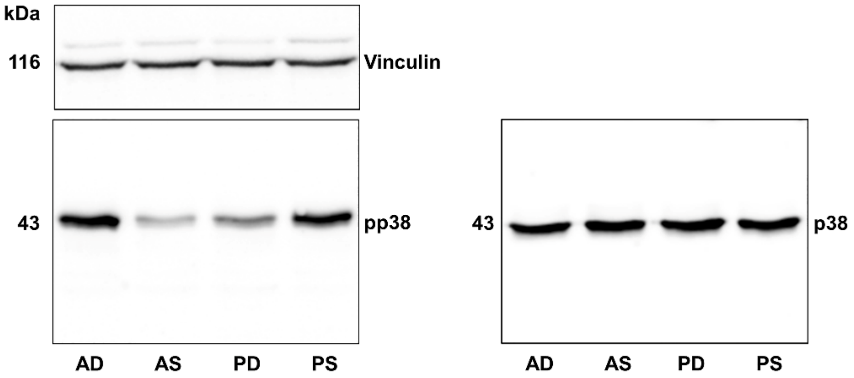

b **Uncropped blot for Fig. 6b**

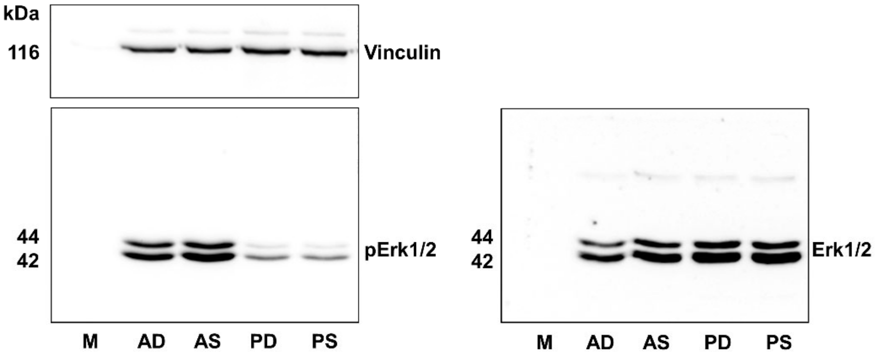

c **Uncropped blot for Fig. 6c**

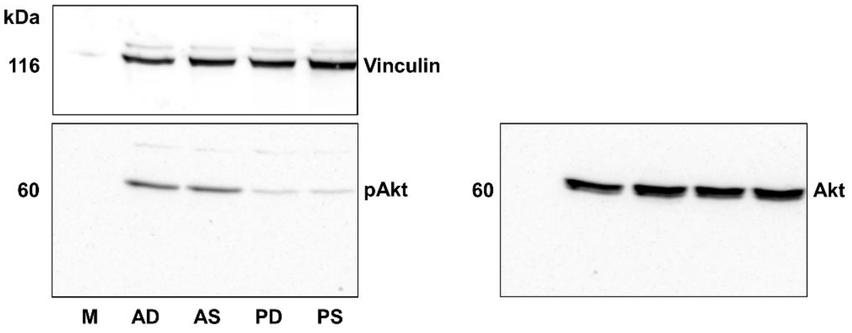

Figure S8. Immunoblot analyses of phospho-p38 (pp38) (**a**), phospho-Erk1/2 (pErk1/2) (**b**), and phospho-Akt (pAkt) (**c**) proteins in whole-cell extracts from AD-, AS-, PD, and PS-HPF cells. Each of the blots was cut at the level of ~80 kDa. The upper part of each blot was developed for the vinculin loading control. The lower part of each blot was first developed for the respective phosphorylated protein. After stripping in buffer containing 62.5 mM Tris-HCl pH 6.8, 100 mM 2-mercaptoetanol, and 2% SDS for 30 min at 50^{0}C, the lower parts were subsequently blocked in 5% w/v BSA, 1x TBS, 0.1% Tween-20, and developed for the respective total proteins used as internal controls. M = protein marker.

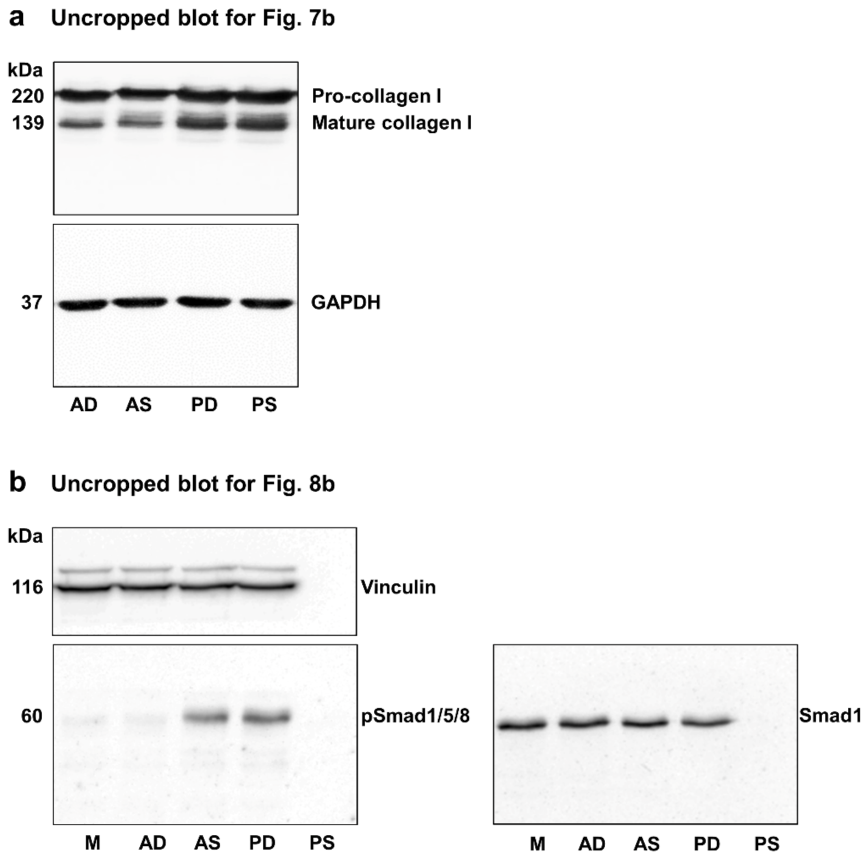

Figure S9. Immunoblot analysis of collagen type I (**a**) and phospho-Smad1/5/8 (pSmad1/5/8) (**b**) proteins in whole-cell extracts from AD-, AS-, PD, and PS-HPF cells. Each of the blots was cut at the level of ~70-80 kDa. The upper part of the blot in (**a**) was developed for collagen type I protein whereas the lower part was developed for the GAPDH loading control. The upper part of the blot in (**b**) was developed for the vinculin loading control whereas the lower part was first developed for the pSmad1/5/8 protein. After stripping in buffer containing 62.5 mM Tris-HCl pH 6.8, 100 mM 2-mercaptoetanol, and 2% SDS for 30 min at 50^0C, the lower part in (**b**) was subsequently blocked in 5% w/v BSA, 1x TBS, 0.1% Tween-20, and developed for the total Smad1 protein used as an internal control. M = protein marker.

Table S1: Primer sequences for genes belonging to the gene sets specifically upregulated in AD- versus PD- as well as in PS- versus PD-CTGs, and related to cell migration and immunomodulation.

Gene symbol	Gene name and accession number	Primer pair (fwd/rev)
CCL2	C-C motif chemokine ligand 2; NM_002982	5′-CAGCCAGATGCAATCAATGCC-3' 5′-TGGAATCCTGAACCCACTTCT-3'
CCL8	C-C motif chemokine ligand 8; NM_005623	5′-GACTTGCTCAGCCAGATTCAG-3' 5′-GTTTGGTCTTGAAGATCACAGCT-3'
CCL24	C-C motif chemokine ligand 24; NM_002991	5′-GGAGTGGGTCCAGAGGTACAT-3' 5′-CAGGTGGTTTGGTTGCCAG-3'
CCL26	C-C motif chemokine ligand 26; NM_001371936.1	5′-CACGTGGGAGTGACATATCC-3' 5′-TTTGGTAGTGAATATCACAGCC-3'
CXCL10	C-X-C motif chemokine ligand 10; NM_001565	5′- TGGCATTCAAGGAGTACCTC-3' 5′- CATTGTAGCAATGATCTCAACACG-3'
CXCL11	C-X-C motif chemokine ligand 11; NM_005409	5′-GACGCTGTCTTTGCATAGGC-3' 5′-GGATTTAGGCATCGTTGTCCTTT-3'
CXCL13	C-X-C motif chemokine ligand 13; NM_006419	5′-GCTTGAGGTGTAGATGTGTCC-3' 5′-CCCACGGGGCAAGATTTGAA-3'
GZMB	granzyme B; NM_001346011.2	5′-TTAAGGGGGACTCTGGAGG-3' 5′-CGTCCATAGGAGACAATGC-3'
CYP1A1	cytochrome P450, family 1, subfamily A, polypeptide 1; NM_000499	5′-TCGGCCACGGAGTTTCTTC-3' 5′-GGTCAGCATGTGCCCAATCA-3'
GAPDH*	glyceraldehyde-3-phosphate dehydrogenase; NM_001256799.2	5′-ATCAAGAAGGTGGTGAAGCAG-3' 5′-TCGTTGTCATACCAGGAAATGAG-3'

*reference gene used for normalization in all qPCR analyses

Table S2: Primer sequences for genes belonging to the gene sets specifically upregulated in A- versus P- as well as in AD- versus PD-CTGs, and related to Erk and Akt signaling.

Gene symbol	Gene name and accession number	Primer pair (fwd/rev)
EGF	epidermal growth factor; NM_001178130.2	5′-TCGGCCACGGAGTTTCTTC-3' 5′-GGTCAGCATGTGCCCAATCA-3'
HBEGF	heparin binding EGF like growth factor; NM_001945	5′-ATCGTGGGGCTTCTCATGTTT-3' 5′-TTAGTCATGCCCAACTTCACTTT-3'

FGF1	fibroblast growth factor 1; NM_000800	5′-ACACCGACGGGCTTTTATACG-3'<br>5′-CCCATTCTTCTTGAGGCCAAC-3'
FGF9	fibroblast growth factor 9; NM_002010	5′-GGCCTGGTCAGCATTCGAG-3'<br>5′-GTATCGCCTTCCAGTGTCCAC-3'
FGF10	fibroblast growth factor 10; NM_004465	5′-CATGTGCGGAGCTACAATCAC-3'<br>5′-CAGGATGCTGTACGGGCAG-3'
FGF12	fibroblast growth factor 12; NM_021032	5′-CTACACCCTCTTCAATCTAATTCC-3'<br>5′-TTCCCCTTCATGATTTGACC-3'
GPC3	glypican 3; NM_001164619	5′-CCTTTGAAATTGTTGTTCGCCA-3'<br>5′-CCTGGGTTCATTAGCTGGGTA-3'
CEACAM1	CEA cell adhesion molecule 1; NM_001205344	5′-CCAGTCACCTTGAATGTCAC-3'<br>5′-GACTTAGCTCAGTGACTATGATCG-3'
EFEMP1	EGF containing fibulin extracellular matrix protein 1; NM_001039348	5′-CGTGTGCCAAGACATAGACG-3'<br>5′-TGGTACATTCATCTATGTCTACGC-3'
LEP	leptin; NM_000230	5′-TGCCTTCCAGAAACGTGATCC-3'<br>5′-CTCTGTGGAGTAGCCTGAAGC-3'
MYOC	myocilin, trabecular meshwork inducible glucocorticoid response; NM_000261	5′-GGCCACCAAAGCTCGACTC-3'<br>5′-GAGGTTGCTGTAGGCAGTCT-3'
NTRK3	neurotrophic tyrosine kinase, receptor, type 3; NM_002530	5′-GCCAGTATCAACATCACGGAC-3'<br>5′-AGCCGGTTACTTGACAGGTTT-3'

Table S3: Primer sequences for genes belonging to the gene sets specifically upregulated in P- versus A- as well as in PD- versus AD-CTGs, and related to ECM organization and connective tissue development.

Gene symbol	Gene name and accession number	Primer pair (fwd/rev)
COL1A1	collagen type I alpha 1 chain; NM_000088.3	5′-GAAGGGACACAGAGGTTTCAG-3'<br>5′-TAGCACCATCATTTCCACGA-3'
COL1A2	collagen type I alpha 2 chain; NM_000089.3	5′-TGGACCTCCTGGTAATCCTG-3'<br>5′-GCTCACCAACAAGTCCTCTG-3'
COL5A1	collagen type V alpha 1 chain; NM_000093	5′-TACAACGAGCAGGGTATCCAG-3'<br>5′-ACTTGCCATCTGACAGGTTGA-3'
COL5A2	collagen type V alpha 2 chain; NM_000393	5′-GACTGTGCCGACCCTGTAAC-3'<br>5′-CCTGGACGACCACGTATGC-3'
COL10A1	collagen, type X, alpha 1; NM_000493	5′-GGGGCTAAGGGTGAAAGGG-3'<br>5′-GGTCCTCCAACTCCAGGATCA-3'

COL11A1	collagen, type XI, alpha 1; NM_001854	5′-GTCCTCCAGGTCTACAAGGC-3'<br>5′-ACGGAACGGTAACATCAACATAG-3'
COL23A1	collagen, type XXIII, alpha 1; NM_173465	5′-CCTGGCGACACTGGGAAAG-3'<br>5′-CCGTCCACACCGTTCTCTC-3'
COL6A2	collagen type VI alpha 2 chain; NM_058174	5′-CAAGCCTGTCTCGTTTGACCT-3'<br>5′-TGTAGACCTTGCCTTCAGACT-3'
ADAMTS2	ADAM metallopeptidase with thrombospondin type 1 motif, 2; NM_021599	5′-GTGCATGTGGTGTATCGCC-3'<br>5′-AGGACCTCGATGTTGTAGTCA-3'
ADAMTS3	ADAM metallopeptidase with thrombospondin type 1 motif, 3; NM_014243	5′-CCGCTCTGGTAGAGGTTAGGA-3'<br>5′-GCTCAGGGTTGGAAGACACG-3'
PCOLCE	procollagen C-endopeptidase enhancer; NM_002593	5′-ACTGGCCCGAGTCCGATTA-3'<br>5′-ACCGAGTCATAGCGGCAGTA-3'
LOX	lysyl oxidase; NM_001178102	5′-ACCACAGGCGATTTGCATGTA-3'<br>5′-GGCAGTCTATGTCTGCACCA-3'

Table S4: Primer sequences for genes belonging to the gene set specifically upregulated in P- versus A-CTGs and related to osteogenesis.

Gene symbol	Gene name and accession number	Primer pair (fwd/rev)
RUNX2	runt related transcription factor 2; NM_001015051.3	5′-AGACCAACAGAGTCATTTAAGGC-3'<br>5′-GGTGTCACTGTGCTGAAGAG-3'
DLX1	distal-less homeobox 1; NM-001038493	5′-ATGCACTGTTTACACTCGGC-3'<br>5′-TTTCTAAAGCAATAGGCCGCA-3'
GATA3	GATA binding protein 3; NM_001002295	5′- GCCCCTCATTAAGCCCAAG-3'<br>5′- TTGTGGTGGTCTGACAGTTCG-3'
CCN4	cellular communication network factor 4; NM_080838	5′- CAGGAACTGCATAGCCTACAC-3'<br>5′- TGGTACACAGCCAGACACTTC-3'
RSPO2	R-spondin 2; NM_178565	5′- GACGCAGTAAGCGAGCTAGTT-3'<br>5′- ACATCGGCTACACCCATTGTC-3'
VCAN	versican; NM_001126336	5′- GAAGGCTTGTTTGGACGTTGG-3'<br>5′- ACGGAATCCATAAGTCCTGACTC-3'
SPP2	secreted phosphoprotein 2; NM_006944	5′- AGAGGGACTACTATGTGTCCAC-3'<br>5′- GGACTCGTCTGAAATGAGACCAA-3'
SPARC1	secreted protein acidic and cystein rich; NM_003118.3	5′- CGAGTTTGAGAAGGTGTGCAG-3'<br>5′- GATGTATTTGCAAGGCCCGA-3'
ASPN	asporin; NM_017680	5′- AACAAGCTAACGAAGATTCACCC-3'<br>5′- CCCCTGGCTCTATCCCATTATT-3'
